# Iso-*seco*-tanapartholides: Isolation, Synthesis and Biological Evaluation

**DOI:** 10.1002/ejoc.200901016

**Published:** 2009-10-07

**Authors:** Edward F Makiyi, Raquel F M Frade, Tomas Lebl, Ellis G Jaffray, Susan E Cobb, Alan L Harvey, Alexandra M Z Slawin, Ronald T Hay, Nicholas J Westwood

**Affiliations:** [a] School of Chemistry and Biomedical Sciences Research Complex, University of St. AndrewsNorth Haugh, St. Andrews, Fife, KY16 9ST, United Kingdom Fax: +44-1334-462585; [b] Wellcome Trust Centre for Gene Regulation and Expression, College of Life Sciences, University of DundeeDow Street, Dundee, DD1 5EH, United Kingdom; [c] Strathclyde Institute of Pharmacy and Biomedical Sciences, University of Strathclyde27, Taylor Street, Glasgow, G4 ONR, United Kingdom

**Keywords:** Natural products, Total synthesis, Terpenoids, Cleavage reaction, Inflammation

## Abstract

The isolation, identification and total synthesis of two plant-derived inhibitors of the NF-κB signaling pathway from the iso-*seco*-tanapartholide family of natural products is described. A key step in the efficient reaction sequence is a late-stage oxidative cleavage reaction that was carried out in the absence of protecting groups to give the natural products directly. A detailed comparison of the synthetic material with samples of the natural products proved informative. Biological studies on synthetic material confirmed that these compounds act late in the NF-κB signaling pathway. (© Wiley-VCH Verlag GmbH & Co. KGaA, 69451 Weinheim, Germany, 2009)

## Introduction

The search continues for bioactive compounds that can act as leads for drug discovery or as tools for biological studies. Whilst the use of large chemical libraries is of importance in this research area,^1^ approaches based on natural products remain powerful.^2^ Here we describe the total synthesis of plant-derived inhibitors of the NF-κB signaling pathway. This pathway plays a key role in inflammation, immunology and cancer. Compounds that modulate its activity have been linked to the treatment of several diseases.^3^

**Figure 1:**
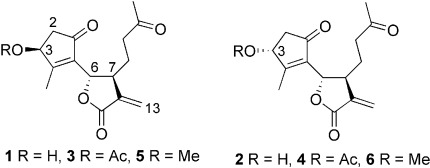
Members of the iso-*seco*-tanapartholide family of natural products isolated from plants of the genus *Artemisia and Achillea*.

In the initial phase, bioactive plant-derived natural-product extracts were identified by using an NF-κB-based reporter-gene assay.^4^ Bioactivity-guided fractionation of one of the extracts resulted in the isolation of what was believed to be the relevant components. Spectroscopic analysis led to structural assignment of the compounds as members of a natural-product family that includes the *seco*-5 and iso-*seco*-tanapartholides (Figure 1).^6^ These interesting structures are believed to be biosynthesised by a Diels–Alderreaction of sesquiterpene lactone derived dienes with oxygen followed by fragmentation of the resulting cyclic peroxides to form the *seco* or iso-*seco* structures.^5^,^7^

The total synthesis of members of this natural-product family was then carried out to confirm their structure and biological activity. A key step in our short and efficient sequence was a late-stage oxidative cleavage reaction that was carried out in the absence of protecting groups to give the natural products directly. A comparison of the previously reported spectroscopic data associated with the natural products proved interesting.

This study was completed by showing that the synthetic samples of the iso-*seco*-tanapartholides are indeed inhibitors of the NF-κB signaling pathway. We also present experiments that have enabled us to propose a mode of action for these inhibitors.

## Results and Discussion

**Figure 2:**
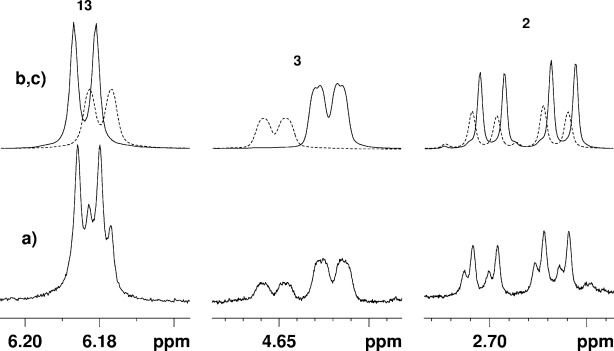
Sections of the ^1^H NMR spectrum of (a) the bioactive sample purified from extracts of the plant *Tanacetum parthenium*; signals corresponding to two compounds are visible for the C3 proton and one each of the C2 and C13 protons; (b) synthetic sample of **1** (dotted line) and (c) synthetic sample of **2** (full line) corresponding to the major compound present in the sample from *Tanacetum parthenium*.

High-throughput screening of plant-derived extracts was carried out by using an HeLa57A cell line that expresses an NF-κB-dependent reporter.^4^,^8^ Failure to trigger this reporter on addition of phorbol 12-myristate 13-acetate indicated that the tested extract inhibited the NF-κB signaling pathway. Whilst several extracts were active, follow-up studies focused on an extract from the plant *Tanacetum parthenium*.^9^ This extract was selected due to the robust nature of the bioactivity observed despite concerns that natural products with the same biological activity have been isolated previously from this plant, including the much studied parthenolide.^10^ Three rounds of bioactivity-guided fractionation resulted in the isolation of an enriched fraction that retained the desired activity.^8^ Detailed spectroscopic analysis of this fraction^8^ led to its assignment as the natural product, iso-*seco*-tanapartholide (reported structure **1**,6a,6b Figure 1), and our plans then focused on how an authentic sample of **1** might be prepared. However, in contrast to previous reports, our analysis showed that the sample of **1** we had isolated was in fact a mixture of two compounds. Figure 2a shows the observed signals assigned to the C3 proton and one each of the C2 and C13 protons and clearly shows that two signals are present for each proton. The two compounds present in the purified extract from *Tanacetum parthenium* were tentatively assigned the epimeric structures **1** and **2** (Figure 1).

Spectroscopic methods alone could not be used to assign the stereochemistry at C3 in the two proposed structures. The remote nature of the C3 stereocenter and the flexibility inherent in this structure contribute to the analysis challenges, as does the fact that it was not possible in our hands to obtain pure samples of the two epimers at this stage. This raised additional concerns about the existing structural assignments within the iso-*seco*-tanapartholide family and provided a further motivation for preparing authentic samples of both **1** and **2**.

**Figure 3:**
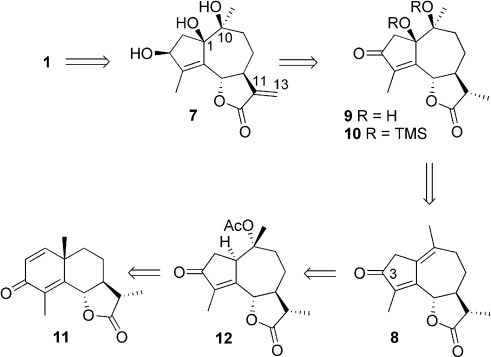
Retrosynthetic analysis of iso-*seco*-tanapartholide.

Retrosynthetically, we envisaged accessing the *seco* structure in **1** by oxidative cleavage of the C1,C10 *syn*-diol functionality in **7** in the absence of protection of the C3 alcohol and αβ-unsaturated lactone functional groups (Figure 3). We reasoned that if the oxidative cleavage reaction was carried out at an earlier stage in the synthesis, a considerable increase in the total number of steps would be required. It was therefore planned that the *syn*-diol unit in **7** would be introduced by dihydroxylation of the γ,δ-double bond in **8**. This dihydroxylation was expected to proceed with high selectivity for the β-face of **8** providing the β-*syn*-diol **9** (Figure 3). This assumption was based on other work from our laboratory in which epoxidation of **8** with *m*-CPBA furnished almost exclusively the β-epoxide.^8^ With β-*syn*-diol **9** in hand it was expected that diastereoselective reduction of the C3 ketone and installation of the C11–C13 *exo*-methylene group would lead to substrates suitable for oxidative cleavage studies.

**Scheme 1:**
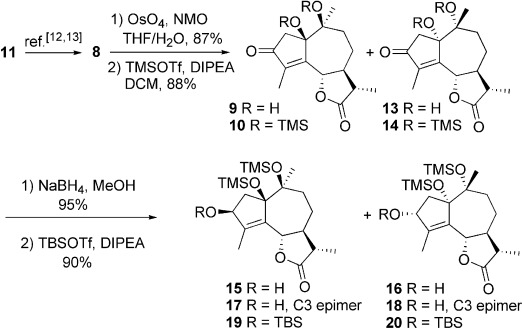
Dihydroxylation and reduction of the C3 carbonyl group.

Our initial plan involved the use of **9** directly in the C3 carbonyl reduction reaction (Scheme 1).^11^ However, for practical reasons, we were forced to revise this by carrying out the reduction on a protected version of **9**, the β-*syn*-diol **10**. Reduction of the C3 carbonyl group in **10** was expected to occur under steric control with approach of the reducing agent occurring from the opposite face to the two protected alcohols giving **7** after removal of the silyl groups. As pure samples of both **1** and **2** were required, it was proposed that epimerisation at C3 by using Mitsonobu protocols would be possible late in the synthesis; however, this proved unnecessary. It was planned to access the 5,7,5 guaianolide system in **8** by photochemical rearrangement of the eudesmanolide (–)-α-santonin **11** to give **12** (Figure 3).^12^ The overall plan, if successful, would provide rapid access to authentic samples of both **1** and **2**.

In practice, acid-mediated elimination of acetic acid from **12**,^13^ obtained from **11**,^12^ provided **8** in accordance with literature precedent. Subsequent dihydroxylation of **8** gave *syn*-diol **9** as the major component in a 3:1 mixture with **13**, with the reaction occurring exclusively at the γ,δ-double bond in **8**. For ease of handling, the diol functionality in both **9** and **13** was protected before reduction of the C3 carbonyl group. Attempts to protect the diol unit in **9** and **13** as acetonides proved unsuccessful returning only the starting materials. This lack of reactivity could be rationalised when the X-ray structures of subsequent intermediates were obtained.^14^ In the cases of **15**–**17**, the seven-membered ring adopts conformations in which the hydroxy groups are positioned such that the O1–C1–C10–O10 torsional angles are 45°, 74° and 55°, respectively. The relatively rigid nature of this framework presumably stops the oxygen atoms moving from their ground state positions to adopt the required conformation for successful acetonisation. However, protection of **9** and **13** was achieved by treatment with TMSOTf yielding a 3:1 mixture of bis(TMS) ethers **10** and **14**. Reaction of **10** and **14** with sodium borohydride provided two major isomers **15** and **16** that, despite being difficult to separate from each other, were readily separable from minor quantities of the other two possible isomers **17** and **18** (ratio **15**+**16**/**17**+**18** = 85:15; 95 % yield). When this purification was repeated on a larger scale, pure samples of **15**, **16** and **17** were isolated and their structures assigned by X-ray analysis. Alcohol **15**, the major product from the reduction of **10**, and likewise alcohol **16**, the major product from the reduction of **14**, result from hydride addition to the less hindered face of the respective ketones, as expected. As **15** and **16** could not be separated easily, reaction of the mixture with TBSOTf resulted in trisilyl ethers **19** and **20** that were readily separable. The pure diastereoisomers **19** and **20** were then carried through the subsequent steps independently.

**Scheme 2:**
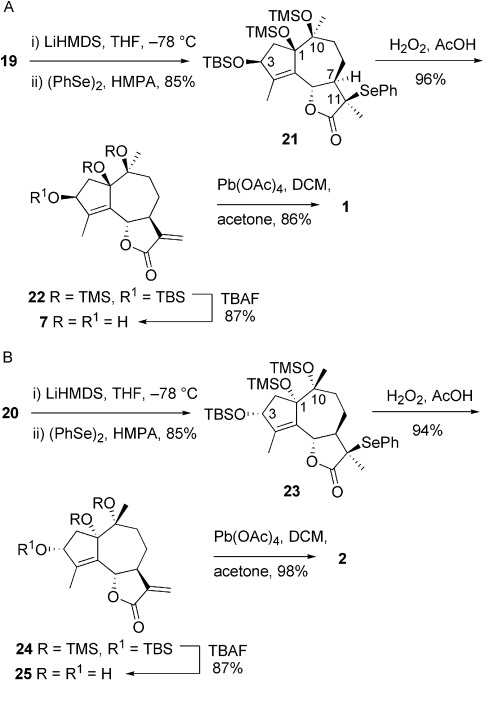
Installation of the exocyclic double bond, global deprotection and oxidative cleavage.

The next challenge was to introduce the exocyclic α-methylene group. Reaction of the lithium enolate generated from **19** with diphenyl diselenide gave exclusively **21** (Scheme 2A).^15^ The stereochemical outcome of this reaction results from pseudoaxial approach of the electrophile and was confirmed by nOe studies on **21**.^8^ Oxidation of the selenide in **21** with concomitant elimination gave **22** as the only product due to the *anti* relationship between the C11 selenoxide and the C7 proton preventing formation of the C7–C11 double bond (Scheme 2A). Global desilylation by using TBAF gave triol **7** in excellent yield, and – pleasingly – **7** was then converted into **1** in 86 % yield on treatment with lead tetraacetate at 0 °C in dichloromethane. Interestingly, attempts to carry out this transformation by using sodium periodate returned only starting material, probably due to the fact that again the O1–C1–C10–O10 torsional angle in **7** is too large and inflexible to enable formation of the required cyclic periodate ester. Whilst lead tetraacetate is known to cleave diols that cannot form the corresponding cyclic ester intermediate, the rates of these reactions are extremely slow.^16^ Therefore, as cleavage of **7** is complete within just 20 min, this reaction probably occurs via the cyclic intermediate that can be formed in this case due to the larger size of lead compared to iodine.^16^ When the reaction of **7** with lead tetraacetate was run for extended times or at higher temperatures over-oxidation of **1** was observed.^17^

In an analogous sequence of reactions, the trisilyl-protected triol **20** (Scheme 1) was transformed via the selenide **23**^8^ into **24**, a compound that contained the required exocyclic α-methylene group (Scheme 2B). Subsequent desilylation of **24** gave **25** which, in an analogous manner to the conversion of **7** into **1**, underwent rapid and clean oxidative cleavage to yield **2** (72 % over 4 steps). Comparison of the ^1^H and ^13^C NMR spectra of synthetic **1** and **2** with that reported for iso-*seco*-tanapartholide isolated from plants of the genus *Artemisia*^6^ proved interesting. The spectra for both **1** and **2** correlated very closely with those reported for the natural product. In the absence of authentic material from these sources, further insight was gained by comparing the optical rotation of the natural material^6^ with those of synthetic **1** and **2**.^18^ Despite the small values involved, it appears that the relative and absolute stereochemistry assigned to iso-*seco*-tanapartholide from *Artemisia* was correct (as in **1**).

However, the situation was complicated when a comparison of synthetic **1** and **2** with our material from *T. parthenium* and a sample from a plant of the genus *Achillea*^19^ was carried out. NMR studies (Figure 2) showed that the bioactive material we had isolated from *T. parthenium* was indeed a mixture of the two epimers **1** and **2** with the major isomer isolated from this plant having the same relative stereochemistry as **2** [compare Figure 2a and c (full line)]. In addition, comparison of our authentic samples of **1** and **2**, with material isolated from a plant of the genus *Achillea*^19^ gave an analogous result, again confirming that the major isomer present in this sample was *epi*-iso-*seco*-tanapartholide **2**. Further optical rotation comparisons^8^,^18^ also supported our conclusion that the major isomer isolated from *Achillea* had the same absolute stereochemistry as **2**. For a more detailed comparison of synthetic **1** and **2** with the previous literature reports of their isolation, see Supporting Information.

**Figure 4:**
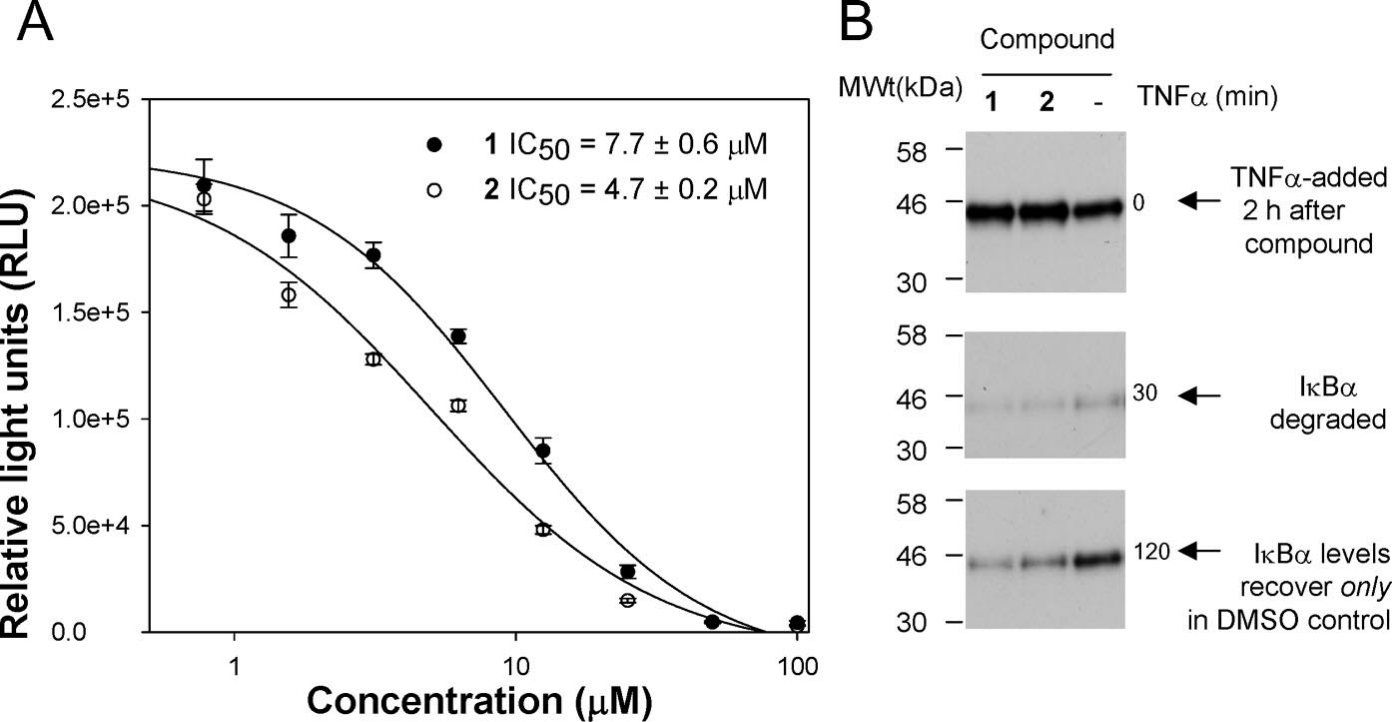
Bioactivity of **1** and **2**: (a) NF-κB-dependent reporter gene assay; (b) IκBα western blot analysis after TNFα activation.

Biological analysis^4^,^20^ of synthetic **1** and **2** confirmed that they inhibited the TNFα activation of NF-κB [IC_50_(**1**) = 7.7 ± 0.6 μm; IC_50_(**2**) = 4.7 ± 0.2 μm; Figure 4A] over a concentration range similar to that observed for our isolated material.^8^ To establish the mode of inhibition, cells were treated with the NF-κB activator TNFα and levels of the NF-κB inhibitor IκBα determined by western blotting. In response to TNFα, IκBα is rapidly degraded and then resynthesized as the IκBα gene is NF-κB-dependent (Figure 4B; DMSO vector). However, in the presence of **1** and **2**, IκBα was degraded, but resynthesis of IκBα was not observed. This indicates that **1** and **2** do not prevent IκBα degradation but block the transcriptional activity of NF-κB. Immunofluorescence studies were also consistent with this assumption.^8^ In addition, we determined the effect of **1** and **2** on the DNA binding activity of NF-κB. Recombinant-purified DNA-binding domains of the p50 and p65 subunits of NF-κB were incubated with **1** and **2** and a gel electrophoresis DNA-binding assay performed.^8^ Both **1** and **2** inhibited NF-κB-DNA binding in a dose-dependent fashion only when a thiol-based reducing agent was absent from the assay. The observed loss of biological activity of **1** and **2** in the presence of a thiol is consistent with a mode of action for these compounds in which covalent modification of cysteine residues in either the p50 or p65 subunit of NF-κB occurs. In addition, a close analogue of **1** in which the exocyclic α-methylene group was replaced by a methyl group [C11-(*S*)] did not inhibit TNFα activation of NF-κB, again suggestive of a role for the exocyclic α-methylene group in **1** as an electrophile. Interestingly, covalent modification of cysteine by inhibitors of NF-κB activation, including the sesquiterpene lactones, has been proposed previously.4b,^21^ In particular, a computational model of the proposed covalent binding mode of the sesquiterpene lactone, helenalin, to Cys38 and Cys120 of the p65 subunit of NF-κB has been described.^22^ Attempts to overlap the structure of the iso-*seco*-tanapartholides onto this model suggest that the C3 functional group would be expected to point away from the protein. If correct, this would provide an explanation for the observed disruption of the NF-κB-DNA interaction by both **1** and **2**, in a manner that is independent of the C3 stereochemistry.

## Conclusions

The bioactivity-guided fractionation of an extract from *Tanacetum parthenium* by using an NF-κB-dependent luciferase reporter gene assay is described. The purified extract was shown to contain two natural products from the iso-*seco*-tanapartholide family. Synthesis of authentic samples of these two natural products by using an efficient oxidative cleavage reaction clarified the structures of the compounds isolated from a series of plants. This resulted in the isolation and synthesis of the natural product *epi*-iso-*seco*-tanapartholide (**2**) as well as iso-*seco*-tanapartholide (**1**). Biological studies on synthetic material confirmed that these compounds act late in the NF-κB signaling pathway.

**Supporting Information** (see footnote on the first page of this article): Details of the Strathclyde natural-product extract collection, screening results, bioactivity-guided purification, structural assignment, experimental procedures, characterisation data for all new compounds, comparison studies of synthetic **1** and **2** with previously reported natural products and material isolated from *T. parthenium* and *Achillea*.

## Supplementary material

Detailed facts of importance to specialist readers are published as ”Supporting Information”. Such documents are peer-reviewed, but not copy-edited or typeset. They are made available as submitted by the authors.
