# Supplementary material for: Iso-seco-tanapartholides: Isolation, Synthesis and Biological Evaluation
Source: European J Org Chem. 2009 Oct 7;2009(33):5711–5. doi: 10.1002/ejoc.200901016 (PMC3627315; doi:10.1002/ejoc.200901016)
Supplement: Supplementary file 1 [file ejoc2009-5711-SD1.pdf]

Eur. J. Org. Chem. **2009** · © WILEY-VCH Verlag GmbH & Co. KGaA, 69451 Weinheim, 2009 · ISSN 1434–193X

SUPPORTING INFORMATION

Title: Iso-*seco*-tanapartholides: Isolation, Synthesis and Biological Evaluation
Author(s): Edward F. Makiyi, Raquel F. M. Frade, Tomas Lebl, Ellis G. Jaffray, Susan E. Cobb, Alan L. Harvey, Alexandra M. Z. Slawin, Ronald T. Hay,* Nicholas J. Westwood*
Ref. No.: O200901016

Page S1 Contents.
Page S2 The Strathclyde natural product extract collection.
Page S2 High-throughput screening.
Page S2-5 Fractionation of the crude extract #2335 from *Tanacetum parthenium*.
Page S5-13 Identification of the active compound.
Page S13-14 Epoxidation of alkene **8**
Page S14-15 nOe Analysis for the selenation products **21** and **23**.
Page S16-18 Comparison of data for synthetic **1** and **2** with previous literature reports.
Page S18-19 Comparison of data for synthetic **1** and **2** with our sample isolated from *Tanacetum parthenium* (Fraction 26 from purification of extract #2335).
Page S20-21 Comparison of data for synthetic **1** and **2** with the sample isolated from *Achillea*
Page S22 General Experimental Procedures
Page S23-33 Experimental protocols.
Page S34-35 Protocols for the biological experiments
Page S36 References

The Strathclyde natural product extract collection.
The collection used in these studies came from the Strathclyde Institute for Drug Research (SIDR). This library contains extracts from a diverse range of plant material, specifically selected to provide maximum species diversity. Plants were collected, dried quickly and material ground into smaller particles and solvent extracted in methanol:tetrahydrofuran (1:5). These crude extracts were then concentrated *in vacuo* and redissolved in DMSO to form the stock solutions that were screened.

High-throughput screening.
Screening was carried out with the HeLa 57A cell line that expresses an NF-κB dependent-luciferase reporter.[S1] In the presence of an inducer, NF-κB is released from IκBα, translocates to the nucleus and activates transcription from the integrated luciferase promoter. Thus, luciferase mRNA levels rise and lead to an increase in luciferase protein. The luciferase expression levels are dependent on the availability of nuclear and active NF-κB that is able to bind DNA and activate transcription. Luciferase activity can be assayed by the emission of light, in the presence of the enzyme substrate (luciferin) and other factors necessary for the reaction to occur. The amount of light emitted can be quantified in a luminometer and values are given in relative light units (RLU). They are proportional to the amount of transcribed luciferase and directly related to the extent of NF-κB-DNA transcriptional activity.
With this purpose, cells grown in 96-well-plates were incubated with the plant material for 2 hours and stimulated with 50ng/ml phorbol 12-myristate-13-acetate (PMA) for 4 hours before being lysed and the amount of luciferase assayed. After preliminary hits had been identified, an additional round of testing was carried out in order to confirm the activity (data not shown). These studies led us to focus on extract #2335, an extract prepared from the plant *Tanacetum parthenium*. A range of bioactive natural products have been isolated previously from this plant including parthenolide, a natural product of relevance to the NF-κB signaling pathway.[S2]

Fractionation of the crude extract #2335 from *Tanacetum parthenium*

Initial purification: The crude extract #2335 was initially purified using a silica column using an elution gradient started at 100% hexane and finishing at 100% DCM in steps of 25%. The material eluted during the first four steps was mixed to constitute fraction 1 and the material eluted with 100% DCM was isolated as fraction 2. A second gradient involving DCM and butanol was then used in which the percentage of DCM decreased from 100% to 75% (fraction 3), to 50% (fraction 4), to 25% (fraction 5) and to 0% ending with 100% butanol (fraction 6). A third gradient was then used between butanol and methanol, where the percentage of butanol decreased from 100% to 75% (fraction 7), to 50% (fraction 8), to 25% (fraction 9) and 0% finishing up with 100% methanol (fraction 10). The fractions were then concentrated *in vacuo* and stored at –20ºC to prevent degradation of the biological material. Fractions were reconstituted in DMSO (1mg dried material in 100μl DMSO) and biologically screened using the HeLa 57A cell line (see above). The results of these studies are shown in Figure S1. Several of the fractions scored in this assay and were progressed to a further purification stage. However, in our hands, the main fraction that consistently led to further fractions that retained the required biological activity was fraction 8.

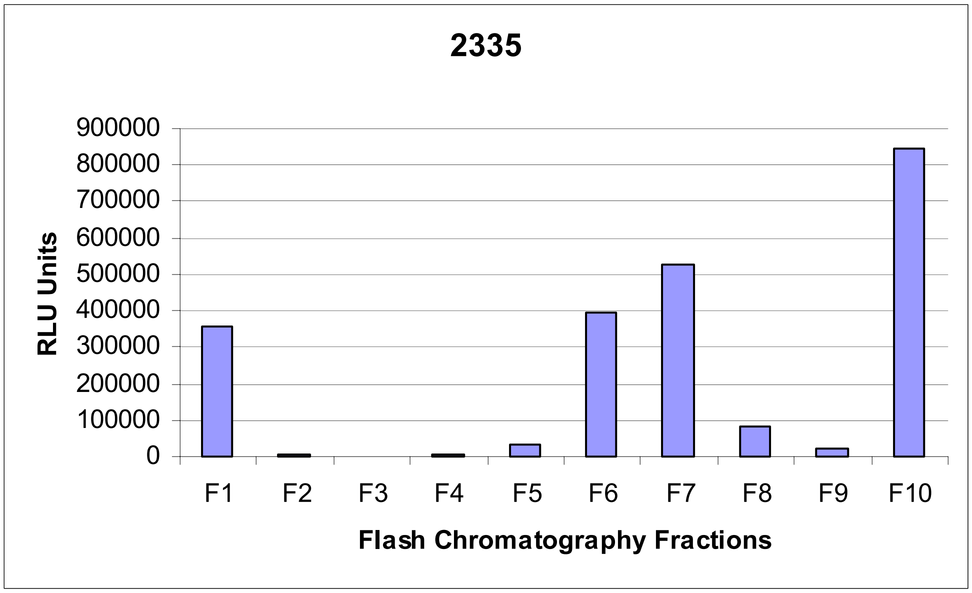

Figure S1. Activity of the fractions isolated from the initial crude purification in the NF-κB dependent-luciferase reporter gene assay. Fraction 8 was selected for further purification.

A dose response curve was generated for fraction 8 prior to further purification (Figure S2). The clear dependence of the biological activity on the concentration of the sample encouraged us to carry out further purification of fraction 8.

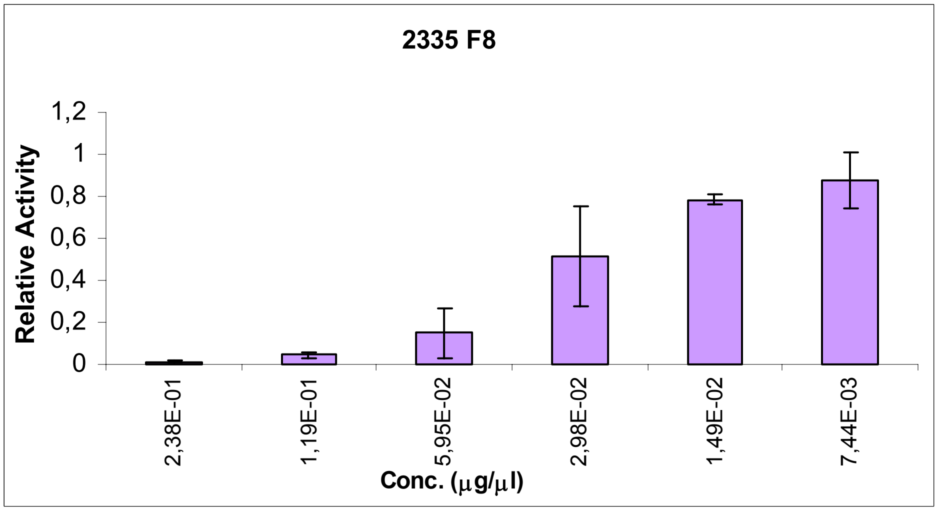

Figure S2: Dose response curve and toxicity evaluation of the plant extract 2335 F8. different concentrations of the extract were incubated with HeLa 57A cells for 2 hours, prior to stimulation with 50 ng/ml PMA for 4 hours. The cells were lysed and luciferase activity was measured in a plate reader luminometer. Results were compared relative to the control sample, where similar amounts of DMSO were used and then represented as the mean ± SD of 2 similar experiments.

At this stage, whilst we had sufficient material in hand, an approximate calculation for the IC_{50} of fraction 8 was also carried out and gave a value of 110 μM (using a MW = 278 Da (for *iso-seco*-tanapartholide) and a concentration of 3 x 10^{-2} μg/μl for the concentration of fraction 8 required to reduce the maximal observed biological readout by 50%). Due to the very limited amount of purified natural product that was eventually isolated this IC_{50} calculation was not repeated on the final material. However, a pure sample of **1** would be expected to have an IC_{50} value that was significantly less than that observed for fraction 8 (as was found to be the case for synthetic **1**). At this stage it was also confirmed using a standard in vitro luciferase assay that fraction 8 did not inhibit the activity of the reporter protein directly (data not shown).

Purification stage 2: Fraction 8 from the fractionated crude extract was repurified using Reverse-Phase High Performance Liquid Chromatography (RP-HPLC). A preparative C18 silica column was used with acetonitrile (ACN), water (H_2O) and 0.1% (v/v) trifluoroacetic acid (TFA) as the mobile phase. 1g of material was purified to produce 53 new fractions of 10ml volume, using a linear gradient from 5% to 75% ACN in H_2O with 0.1% TFA at a flow rate of 10 ml/min. Detection wavelengths of 220 nm, 250 nm and 270 nm were used. Fractions from this run were screened using the HeLa 57A assay (see Figure S3) and fractions 34-41 were selected, concentrated *in vacuo* and prioritized for further purification.

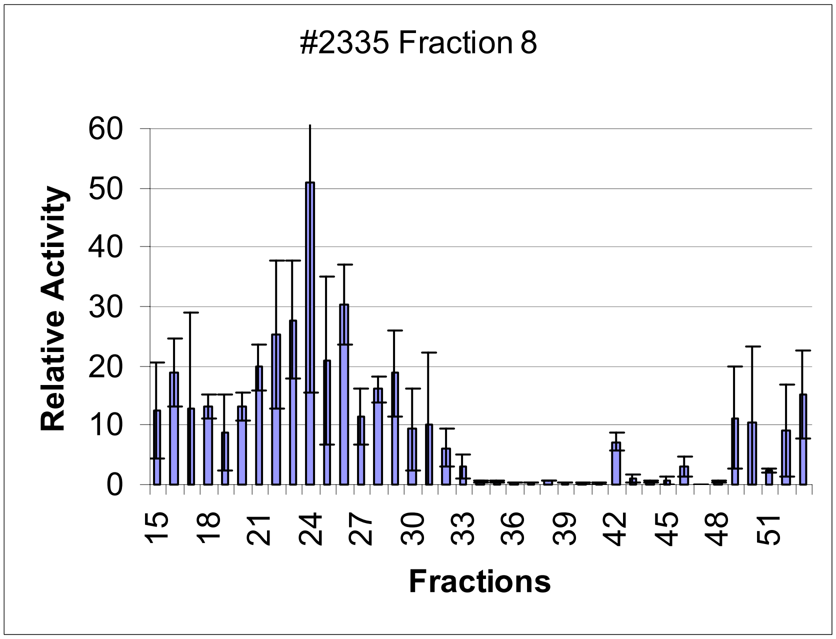

Figure S3. Activity of the fractions isolated from purification stage 2. Fractions 34-41 were combined and carried on to the next stage.

Purification stage 3: In the final round of purification, the combined fractions 34-41 from stage 2 were repurified using a preparative C18 silica column as follows: a first gradient step from 5 % to 20 % ACN (2.0 CV) / a segment step at 20% ACN (1.5 CV) / a gradient step from 20% to 22.5% ACN (1.0 CV) / a segment step at 22.5% ACN (1.5 CV) / a gradient segment from 22.5% to 25% ACN (1.0 CV) and a segment step at 25% ACN (1.5 CV) was used. The flow rate was 1.5 ml/min and 50 new fractions of 4ml volume were collected. Fractions from stage 3 were screened as described previously with fractions 25-36 (Figure S4) all showing

inhibitory activity in the HeLa 57A assay. Fraction 26 was selected for structure identification studies as it was judged to contain significant amounts of material and to be the cleanest.

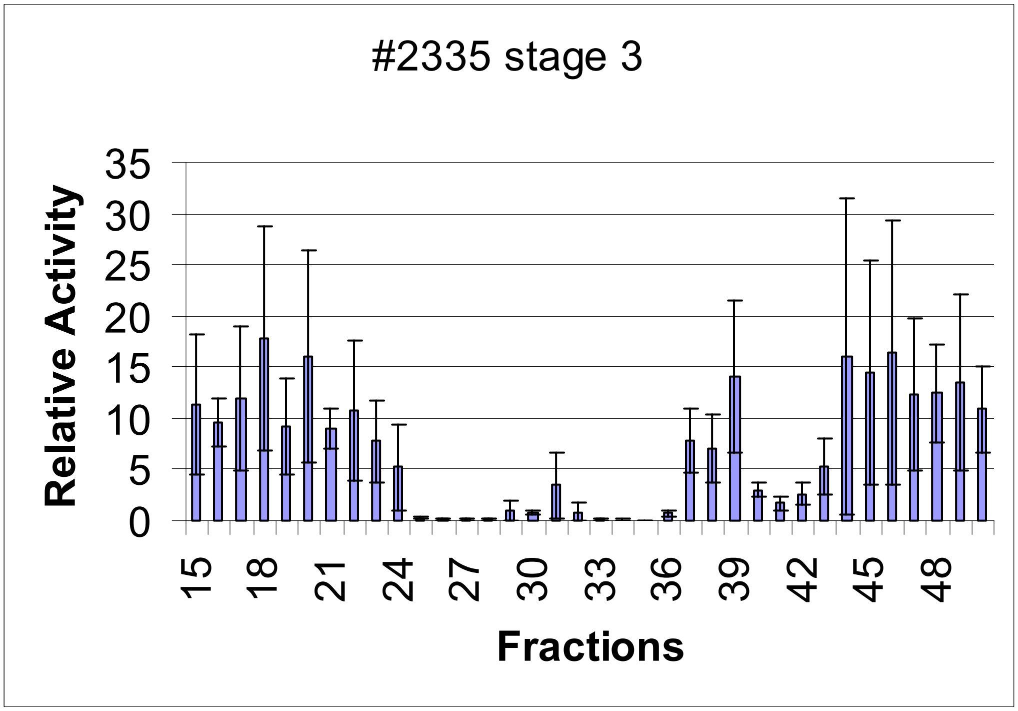

Figure S4. Activity of the fractions isolated from purification stage 3.

Identification of the active compound.

To elucidate the structure of the active compound in the fraction 26 (*RFMF_F26)* 1D ^{1}H (Figure S5), 2D ^{1}H,^{1}H COSY (Figure S6), ^{1}H,^{1}H TOCSY (Figure S7), ^{1}H,^{13}C edited HSQC (Figure S8) and ^{1}H,^{13}C HMBC (Figure S9) spectra were recorded in acetonitrile-d3. All ^{1}H and ^{13}C data obtained from these experiments are summarised in Table S1 and Table S2, respectively.

The TOCSY spectrum (Figure S7) clearly showed that protons H-13, H-6, H-7, H-9 and H8 were part of one extended spin-system whereas the COSY spectrum (Figure S6) indicated that proton H-7 coupled to all the other protons in the spin-system except H9. According to the HSQC spectrum (Figure S8), both protons H-13 were bonded to the same carbon at 122.1 ppm which suggested that they were geminal protons of a C=C double bond. In the HMBC spectrum (Figure S9), long-range correlations were observed from protons H-13 and H-6 to C-12 (δ 171.4) and from H-13 and H-8 to C-11 (δ 140.9). All the above mentioned correlations indicated the presence of a sesquiterpene lactone ring with an aliphatic chain bonded to the carbon C7 adjacent to exocyclic double bond. The HMBC spectrum (Figure S9) also showed two ketone carbonyls, C-10 and C-1 (δ 209.0 and 204.9, respectively). Since the carbonyl C-10 correlates in the HMBC spectrum with protons of CH_2-8, CH_2-9 and CH_3-14 it was

concluded that the aliphatic chain ends with an acetyl group. On the other hand, the carbonyl C-1 correlated with both protons of CH_2-2 and proton H-6. According to the COSY spectrum (Figure S6), protons of CH_2-2 and proton H-3 form a separate ABX spin-system. Moreover, the HMBC spectrum (Figure S9) showed long-range correlations from H3 to C-4 and C-5 (δ 175.6 and 138.0, respectively). Both carbons C-4 and C-5 also correlate with protons of CH_3-15, CH_2-2 and H6. The observation of these correlations enabled us to propose that C-6 of the sesquiterpene lactone ring is substituted by a 3-hydroxy-2-methyl-5-oxocyclopent-1-enyl ring. The overall structure (Figure S10) with a molecular formula of $C_{15}H_{18}O_5$ is also in good accordance with the mass spectroscopic analysis (Figure S11) where peaks were detected at *m*/*z* (M + H^+) = 279, (M + Na^+) = 301 and (M + Na^+ + CH_3CN) = 345.

The relatively small coupling observed between H-6 and H-7 (*J* = 5.7 Hz) did not allow us to draw an unambiguous conclusion concerning the stereochemistry of the lactone ring. Therefore a selective 1D gs-NOESY experiment was carried out (Figure S12). nOe enhancements observed for protons H-9 and H8 after selective inversion of H-6 clearly confirmed the *trans*-configuration which has been reported for all natural products isolated plants of the genus *Artemisia* to date. However, closer inspection of H-2a, H-3 and H-13 resonances in the ^{1}H NMR spectrum revealed that *RFMF_26* consisted of two diastereomers (Figure S13). This conclusion was also apparent from the H-3, C-3 cross-peak in the HSQC spectrum (Figure S14) and the H-3,H-2a and the H-3,H-2b crosspeaks in the COSY spectrum (Figure S15). Since the biggest differences in chemical shifts are observed for H-3 and H2 resonances it seemed very likely that *RFMF_26* fraction contained both C-3 epimers. Attempts to assign the stereochemistry at C3 for the major and minor epimers proved unsuccessful due to the relatively remote nature of this stereocentre and the relative flexibility of this structure.

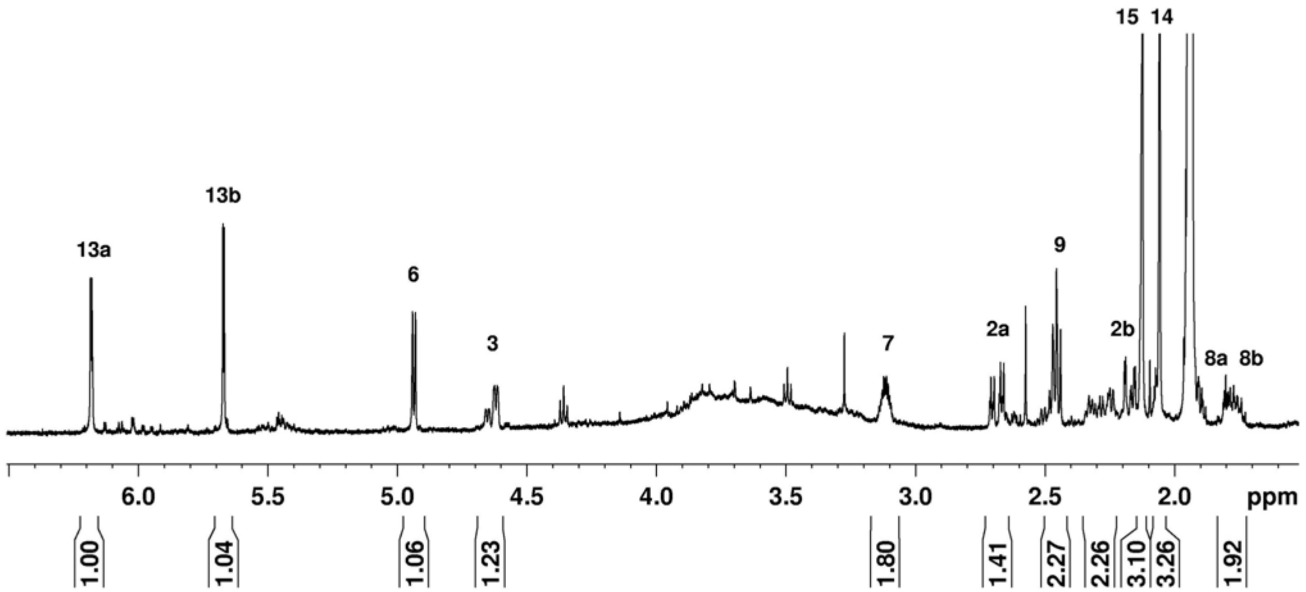

Figure S5 ^{1}H NMR of *RFMF_26* in acetonitrile-d3.

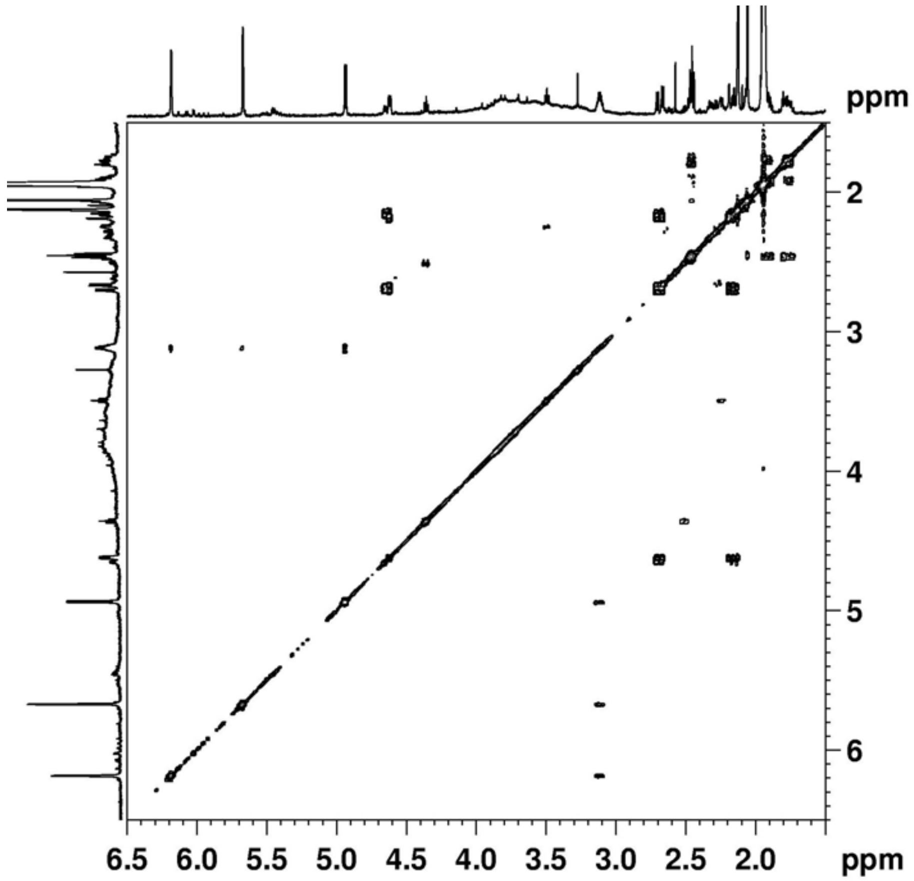

Figure S6 ^{1}H,^{1}H-COSY of *RFMF_26* in acetonitrile-d3.

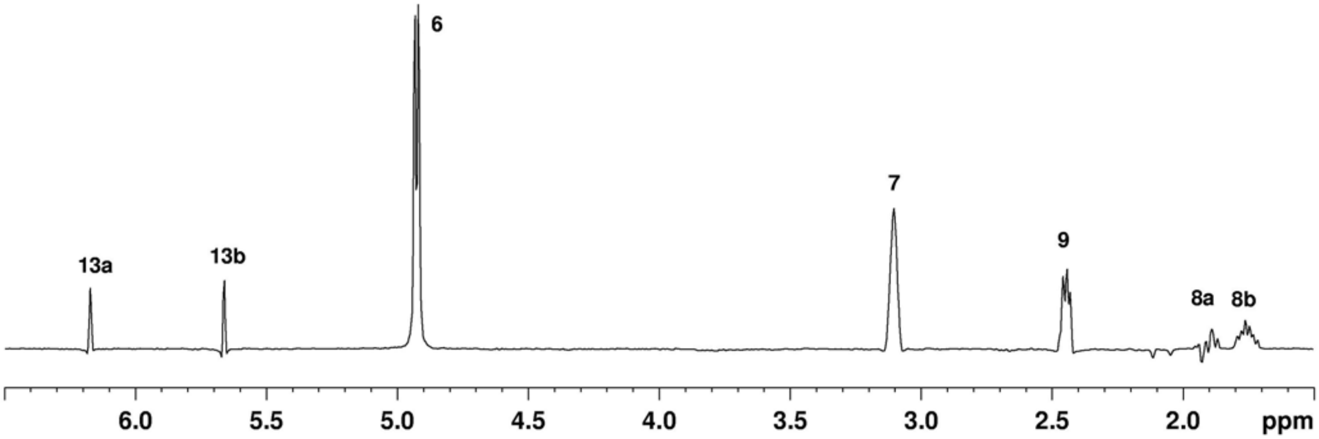

Figure S7 A row taken from 2D ^{1}H,^{1}H TOCSY of *RFMF_26* at 4.93 ppm.

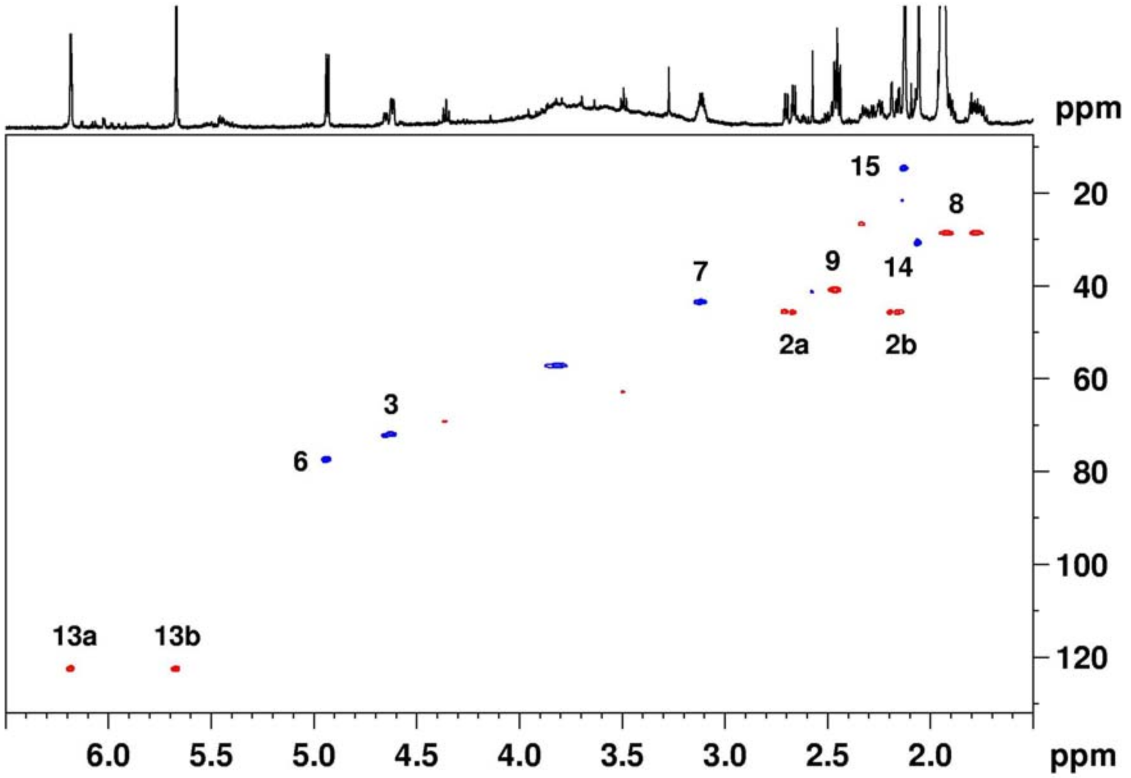

Figure S8 Multiplicity edited $^{1}H,^{13}C$ HSQC of *RFMF_26* in acetonitrile-d3.

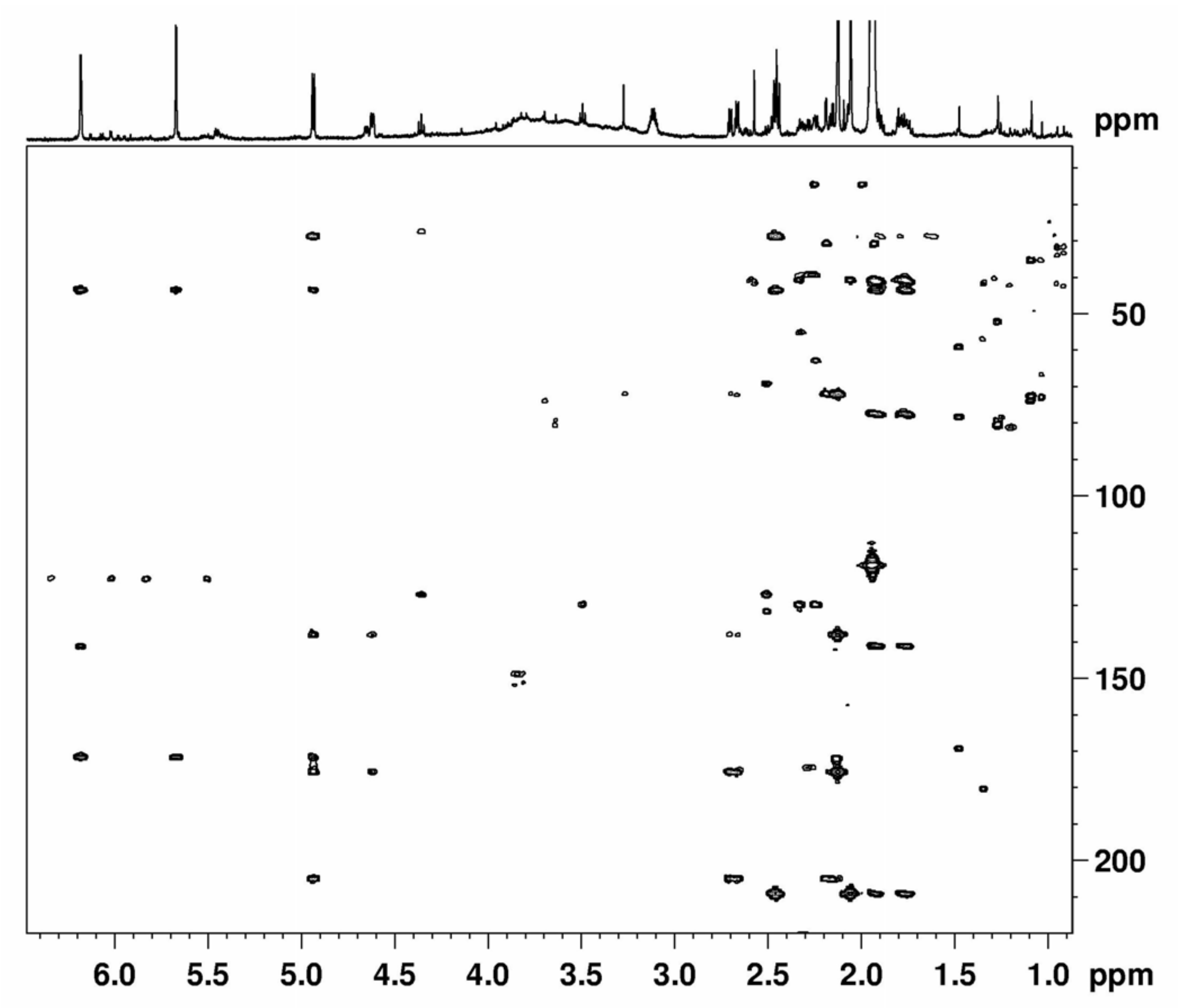

Figure S9 ^{1}H,^{13}C HMBC of *RFMF_26* in acetonitrile-d3.

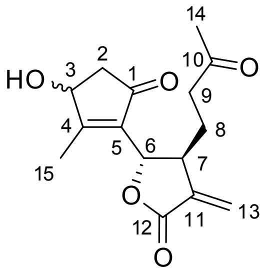

Figure S10 Structure and numbering of the active compounds in *RFMF_26*

Table S1 ^{1}H NMR		
2a	2.17	dd, (18.3, 2.5)
2b	2.68	dd, (18.3, 6.3)
3	4.62 (4.65)	app. broad d (6.3)
6	4.94	d (5.7)
7	3.11	m
8a	1.77	m
8b	1.91	m
9	2.46	dd (6.9, 7.8)
13a	5.67	d (2.6)
13b	6.18	d (3.0)
14	2.06	s
15	2.12	s

Table S2 ^{13}C NMR data	
1	204.9
2	45.4
3	71.8 (72.0)
4	175.6
5	138.0
6	77.2
7	43.4
8	28.4
9	40.8
10	209.0
11	140.9
12	171.4
13	122.1
14	30.5
15	14.4

Table S1 Assignment of ^{1}H NMR spectrum of the active compound in *RFMF_26*. For H3 signal value in parentheses corresponding to the signal assigned to the minor C3-epimer. Coupling constants shown in parentheses in column 3. For the numbering system used see Figure S10.

Table S2 Assignment of ^{13}C NMR spectrum of the active compound in *RFMF_26*. For C3 signal value in parentheses corresponding to the signal assigned to the minor C3-epimer

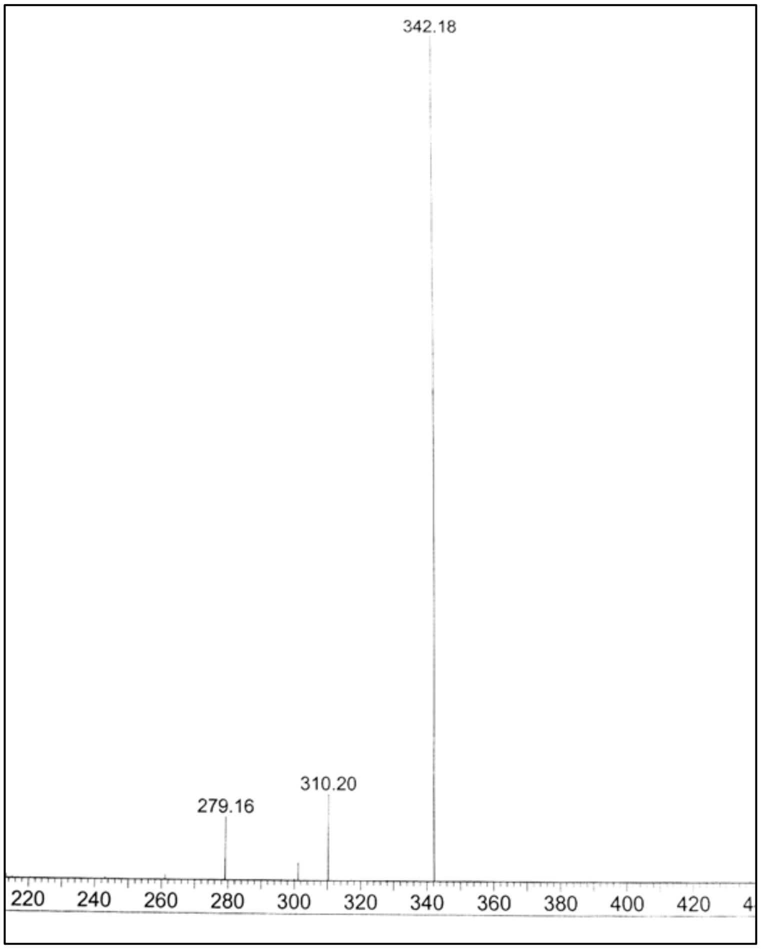

Figure S11: Mass spectrometric analysis of fraction 26 (*RFMF_26*). Positive ion electrospray analysis (cone voltage: 25 volts) of fraction 26 dissolved in acetonitrile. The sample was injected directly into the mass spectrometer.

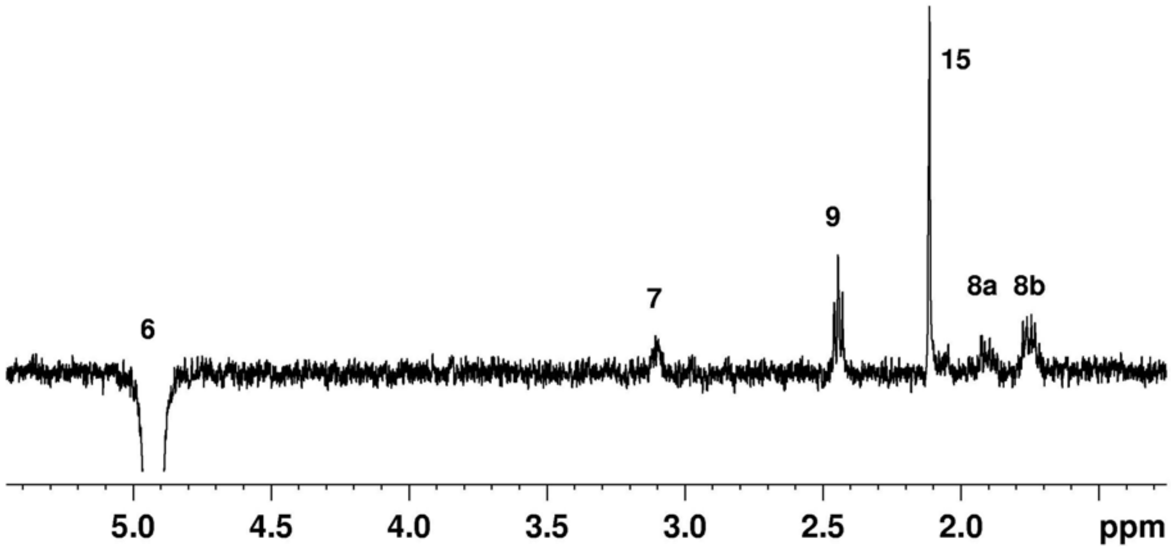

Figure S12 1D gs-NOESY of *RFMF_26* showing nOe enhancements after selective inversion of proton H-6.

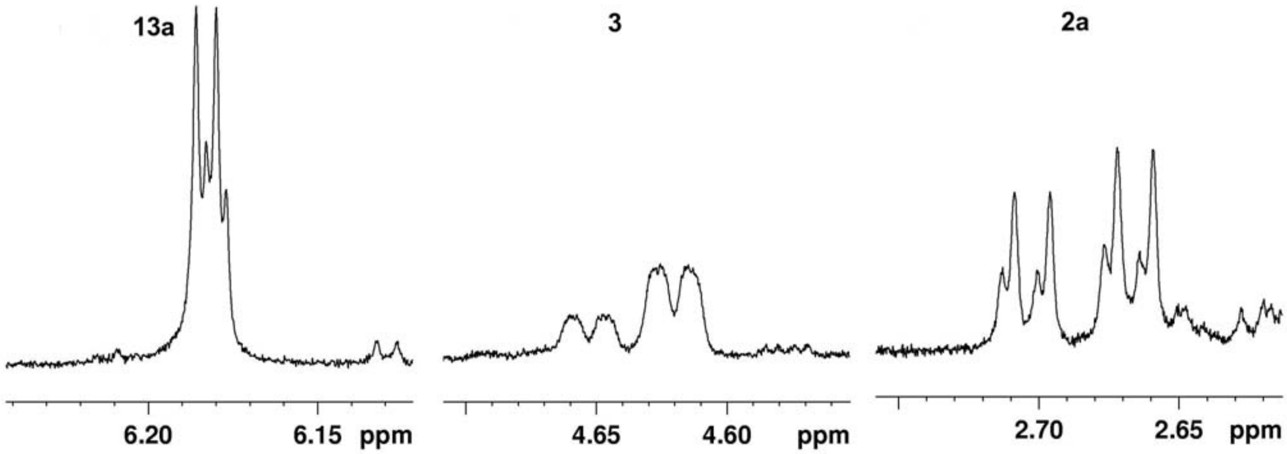

Figure S13 Sections from ^{1}H NMR spectrum of *RFMF_26* showing H-2a, H-3 and H-13a resonances where presence of two epimers is apparent.

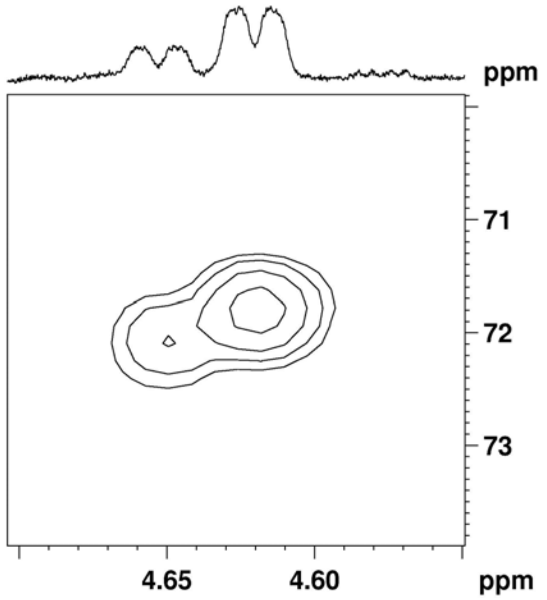	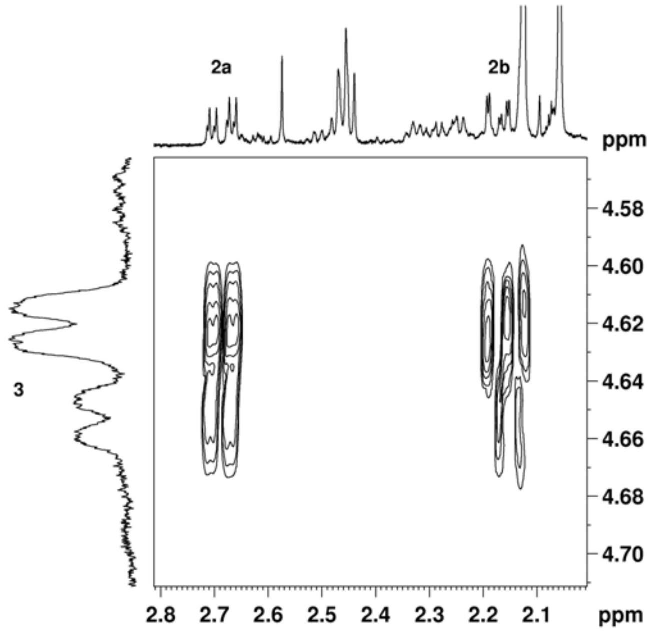
**Figure S14** Section from HSQC spectrum showing H-3,C-3 crosspeak	**Figure S15** Section from COSY spectrum showing H-3,H2a and H-3,H2b crosspeak.

Epoxidation of 8

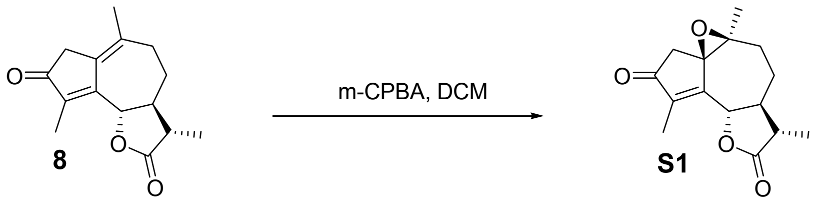

To a solution of **8** (668 mg, 2.71 mmol) in DCM (15 ml) at room temperature was added *m*-CPBA (493 mg, 2.85 mmol) and reaction followed by TLC. Reaction was complete after 6 hr. Saturated aqueous $NaHCO_3$ solution (20 ml) was added and the mixture extracted with DCM (2 x 70 ml). Combined organic phases were washed with water (30 ml), dried and concentrated to give a crude mixture which was purified by flash column chromatography (SiO_4, 6:4 hexanes: ethyl acetate) giving the β-epoxide **S1** as a white solid (711 mg, 85 % yield). X-ray quality crystals of **S1** were obtained by slow evaporation of hexanes/DCM solution of pure **S1** (data submitted to CCDC).

$\mathbf{R}_f$ = 0.20 (6:4 hexane/ ethyl acetate), **mp** 134 – 136 °C, **^{1}HNMR** ($CDCl_3$, 400 MHz) 4.84 (1H, d, *J* = 10.3 Hz, H-6), 2.77 – 2.70 (1H, d, *J* = 18.9 Hz, H-2a), 2.63 – 2.55 (1H, d, *J* = 18.9 Hz, H-2b), 2.40 – 2.26 (2H, m, H-9a, H-11), 2.08 – 1.98 (1H, m, H-9b), 2.03 (3H, s, CH_3-15), 1.90 –

1.82 (1H, m, H-8a), 1.71 – 1.54 (2H, m, H-7, H-8b), 1.43 (3H, s, CH_3-14), 1.28 (3H, d, J = 7.0 Hz, CH_3-13).
^{13}CNMR ($CDCl_3$, 100 MHz) 203.3, 177.3, 159.5, 141.5, 82.2, 68.5, 66.4, 50.1, 41.4, 40.8, 33.5, 25.7, 24.5, 12.6, 9.26. **$[\alpha]^{20}_D$** = -79.3 (c = 0.003 in $CHCl_3$), **HRMS** (TOF ES^+) (m/z) calcd. for $C_{15}H_{18}O_4Na$ ($[M+Na]^+$): 285.1103, found: 285.1102.

nOe Analysis for the selenylation products 21 and 23.

Evidence to support the assigned stereochemistry of compounds **21** and **23**, prepared by selenylation of **19** and **20** respectively came from the use of nOe experiments. The observed enhancements were consistent with the assigned stereochemistry (Figure S12 and S13)

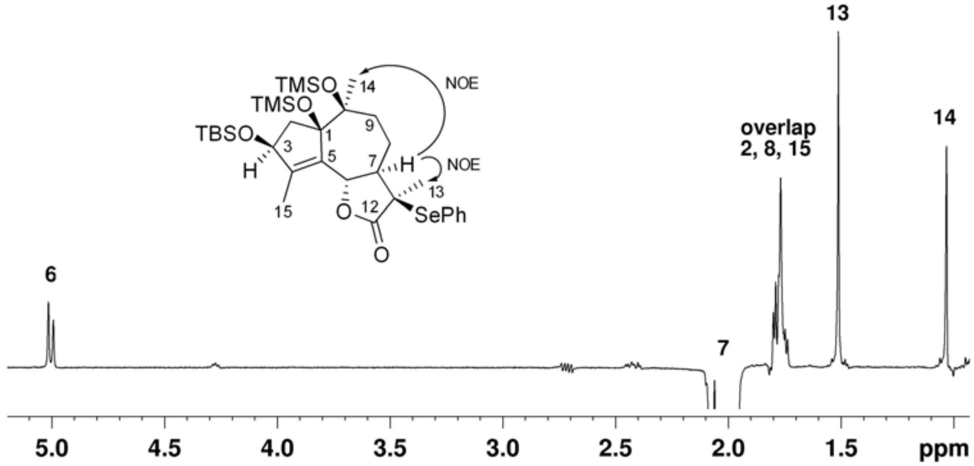

Figure S16 Experiment used to support the stereochemical outcome in the synthesis of **21**.

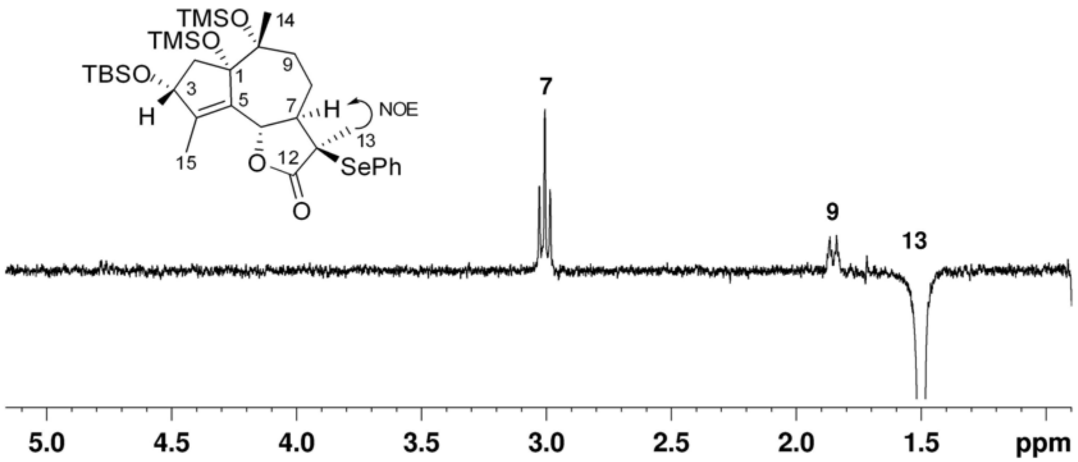

Figure S17 Experiment used to support the stereochemical outcome in the synthesis of **23**.

Comparison of data for synthetic 1 and 2 with previous literature reports.

Upon completion of the synthesis of compounds **1**and **2** we compared our analytical data with that reported in literature. References S3-S11 list the previous reports of the isolation of *iso-seco*-tanapartholide. Huneck and co-workers were the first to name and assign structure **1** to iso-seco-tanapartholide isolated from a plant of the genus *Artemisia*.[S3] They assigned the stereochemistry of the hydroxyl group at C3 as being β (*S*) based on "observed couplings" in the ^{1}H NMR spectrum (400MHz, $CDCl_3$) although details of how this was achieved are missing in their report.[S3] Comparison of the ^{1}H NMR spectra we obtained for our synthetic samples of **1** and **2** with the signals reported by Huneck[S3] (Table S3) showed how similar the spectra of the two isomers are to each other and highlighted the difficulties of doing this comparison with Huneck's report.[S3] Despite the samples being run under analogous conditions, it is not possible to conclude based on the ^{1}H NMR spectrum alone whether Huneck had isolated **1** or its isomer **2**, although it is clear that the basic structure of *iso-seco*-tanapartholide is correct. To date we have been unable to obtain an authentic sample of the material reported by Huneck. An analogous conclusion is also reached when a comparison of the ^{13}C NMR data reported for *iso-seco*-tanapartholide by Marco[S4] (also isolated from a plant of the genus *Artemisia*), is carried out with our data for synthetic **1** and **2** (Table S4). Unfortunately, the sample reported by Marco[S4] has subsequently decomposed (personal communication from Professor Marco).

		**Synthetic 1**<br>400MHz,$CDCl_3$<br><br>C3-β(*S*)-OH	**Synthetic 2**<br>400MHz, $CDCl_3$<br><br>C3-α(*R*)-OH	Huneck 1986 [S3]<br>400MHz, $CDCl_3$<br>assigned as<br>C3-β(*S*)-OH	Todorova 1985 [S12]<br>250MHz,$CDCl_3$
1	Quat				
2	CH_2	2.33dd, 2.79 dd	2.32 dd, 2.79 dd	2.31 dd, 2.82 dd	2.33 d, 2.78 dd
3	CH	4.73 br t	4.70 br t	4.72 br d	4.70 br s
4	Quat				
5	Quat				
6	CH	4.93 d	4.94 d	4.97 d	4.94 d
7	CH	3.14 m	3.09 m	3.09 dddt	3.12 m
8	CH_2	1.86 m, 1.98 m	1.85 m, 1.94 m	1.85 ddt, 1.94 ddt	1.90m
9	CH_2	2.52 m	2.54 m	2.54 dt, 2.59 dt	2.55 m
10	Quat				
11	Quat				
12	Quat				
13	2 x CH	5.66 d, 6.34 d	5.65 d, 6.33 d	5.67 d, 6.35 d	5.67 d, 6.32 d
14	CH_3	2.15 s	2.14 s	2.12 s	2.20 s
15	CH_3	2.18 s	2.17 s	2.14 s	2.15 s
	OH	2.47 d	2.98 br d		3.90 s

Table S3. Comparison of ^{1}H NMR data for synthetic **1** and **2** with existing reports in the literature

Interestingly, the compound isolated and reported by Todorova[S5] in 1985 (structure **S1** below). has very similar spectroscopic analysis to our synthetic **1** and **2** and to the compounds reported by Huneck[S3] and Marco[S4]. It is therefore likely that the compound isolated by

Todorova in 1985 is either **1** or **2** and not the structure reported in that paper. It has not been possible to confirm this due to the absence of authentic material. No further reports concerning a compound with the structure reported by Todorova[S12] exist in the literature to the best of our knowledge.

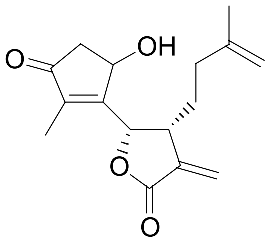

S1

		**1** 100 MHz, $CDCl_3$ C3-β(*S*)-OH	**2** 100 MHz, $CDCl_3$ C3-α(*R*)-OH	Marco 1993 [S4] 75MHz, $CDCl_3$ assigned as C3-β(*S*)-OH	Todorova 1985 [S12] 63MHz, $CDCl_3$
1	quat	203.3	203.4	203.1	203.66
2	CH_2	44.4	44.4	44.3	44.39
3	CH	72	71.7	71.5	71.62
4	quat	172.9	173.4	173	173.77
5	quat	137.7	137.7	138.2	138.46
6	CH	76.4	76.2	76	76.44
7	CH	42.8	42.9	42.9	42.7
8	CH_2	27.6	27.4	27.3	27.48
9	CH_2	39.8	39.6	39.5	39.63
10	quat	207.9	207.9	207.7	207.79
11	quat	138.4	138.4	137.6	137.24
12	quat	170	170.2	170	170.2
13	2 x CH	122.9	123	122.9	122.74
14	CH_3	30.1	30.1	30	29.96
15	CH_3	14.2	14.2	14.1	14.06

Table S4. Comparison of ^{13}C NMR data for for synthetic **1** and **2** with existing reports in the literature

Some clarity regarding the existing literature does come from a comparison of the optical rotations of our synthetic **1** and **2** with the sample reported by Huneck[S3] and Marko[S4] from *Artemisia*. Marco's reported optical rotation for *iso*-*seco*-tanapartholide $[\alpha]^{20}_D$ = +3.3 (c = 0.006 solvent not mentioned)[S4] was in close agreement with the value we obtained for synthetic **1** ($[\alpha]^{20}_D$ = +2.9 (c = 0.008 in $CHCl_3$). Comparison with the value we obtained for synthetic **2**

($[\alpha]^{20}_D$ = -6.5 (c = 0.002 in $CHCl_3$) further supported the fact that the Marco's sample[S3] did not have the structure **2**.

Professor Ryu kindly provided us with a ^1H NMR spectrum for "compound 2" reported in reference S10 (Figure S18). This sample referred to as "compound 2" was isolated from *Artemisia iwayomogi*. Whilst it is interesting to note that this sample is present as a single epimer, in the absence of isolated material and optical rotation data it is not possible to ascertain whether this material corresponds to *iso*-*seco*-tanapartholide **1** or *epi*-*iso*-*seco*-tanapartholide **2**.

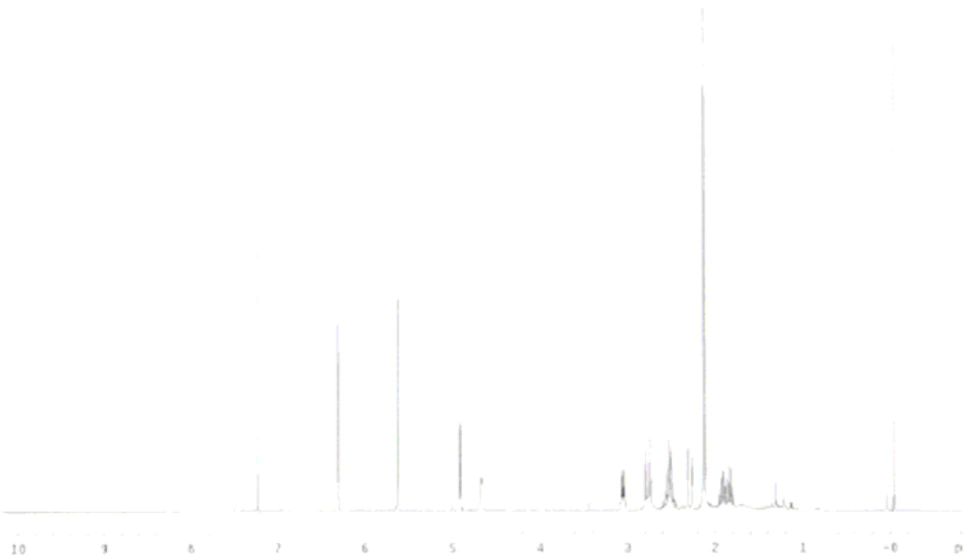

Figure S18. ^1H NMR of the named "compound 2" reported by Ryu et al. ($CDCl_3$, 600MHz)[S10]

Comparison of data for synthetic 1 and 2 with our sample isolated from *Tanacetum parthenium* (Fraction 26 from purification of extract #2335)

In order to confirm our initial conclusion that the material we had isolated was a mixture of diastereoisomers differing only in the configuration at C3, direct comparison of ^1H NMR spectra for our isolated material with our synthetic **1** and **2** was made (Figure S19). The ^1H NMR spectra for synthetic **1** and **2** were rerun in CD_3CN to enable the comparison and as can be seen from Figure S19 all the relevant signals are very closely matched, with the only easily detectable difference in chemical shifts between them being those observed in the ^1H NMR for the C3 proton. Superimposing all three ^1H NMR spectra (isolated material, synthetic **1** and synthetic **2**) exhibited an excellent match and revealed **2** to be the major component in our isolated sample. Unfortunately, due to the very limited amount of material isolated in a pure form, we were unable to obtain an optical rotation.

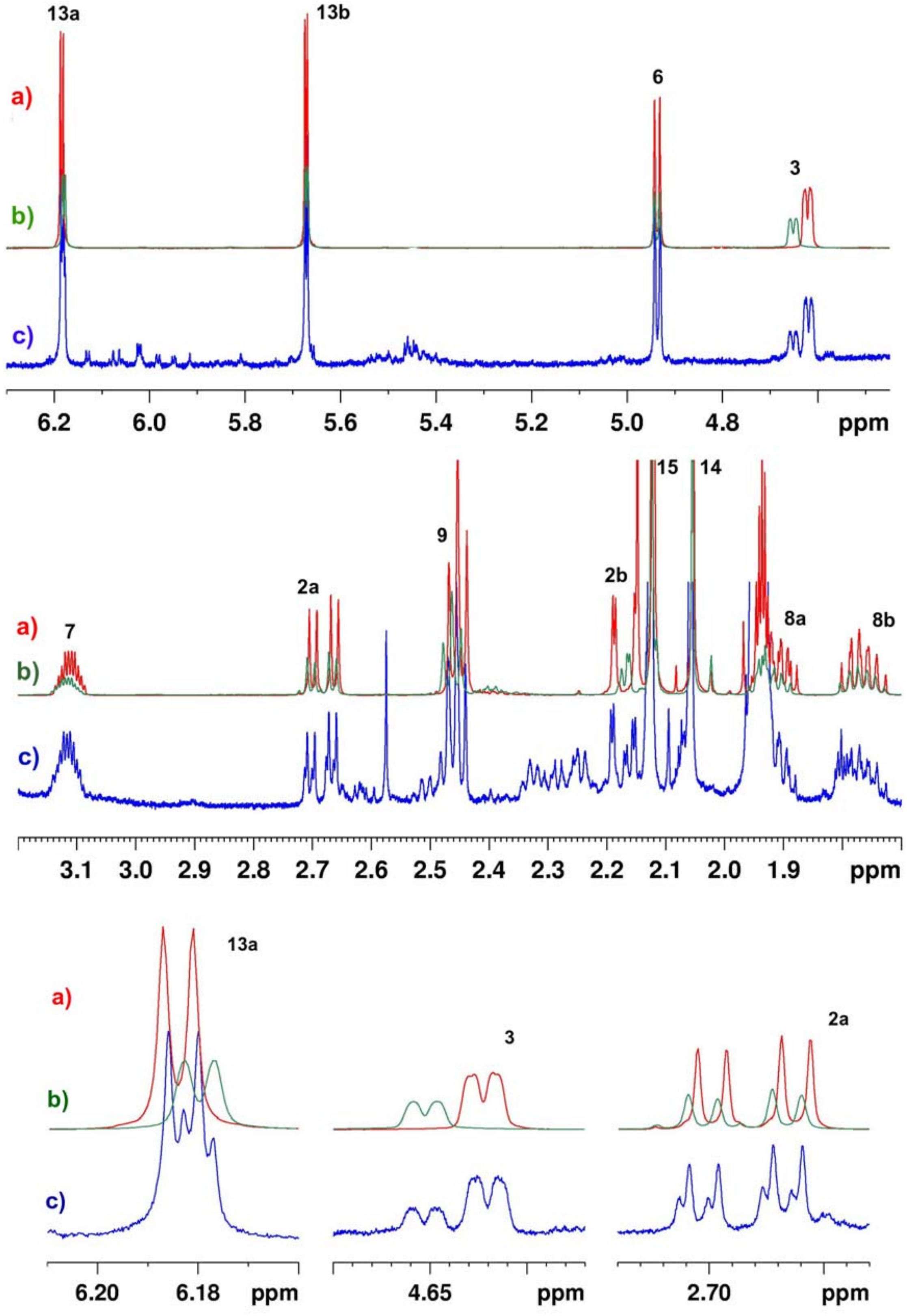

Figure S19 Overlay of ^{1}H NMR spectra (500MHz, CD_3CN) of a) synthetic compound **2** b) synthetic compound **1** c) our isolated material from *T.parthenium.*

Comparison of data for synthetic 1 and 2 with a sample isolated from *Achillea*.[S11]

Professor Todorova kindly provided us with a sample of "*iso*-*seco*-tanapartholide" from a plant of the genus *Achillea*. We carried out a detailed comparison of this material with our synthetic **1** and **2**. This comparison clearly showed (Figure S20) that this sample was a mixture of two epimers with the same relative stereochemistry as **1** and **2**. It also showed that the major isomer present had the same relative stereochemistry as **2**, whilst the minor isomer had the same relative stereochemistry as **1**.

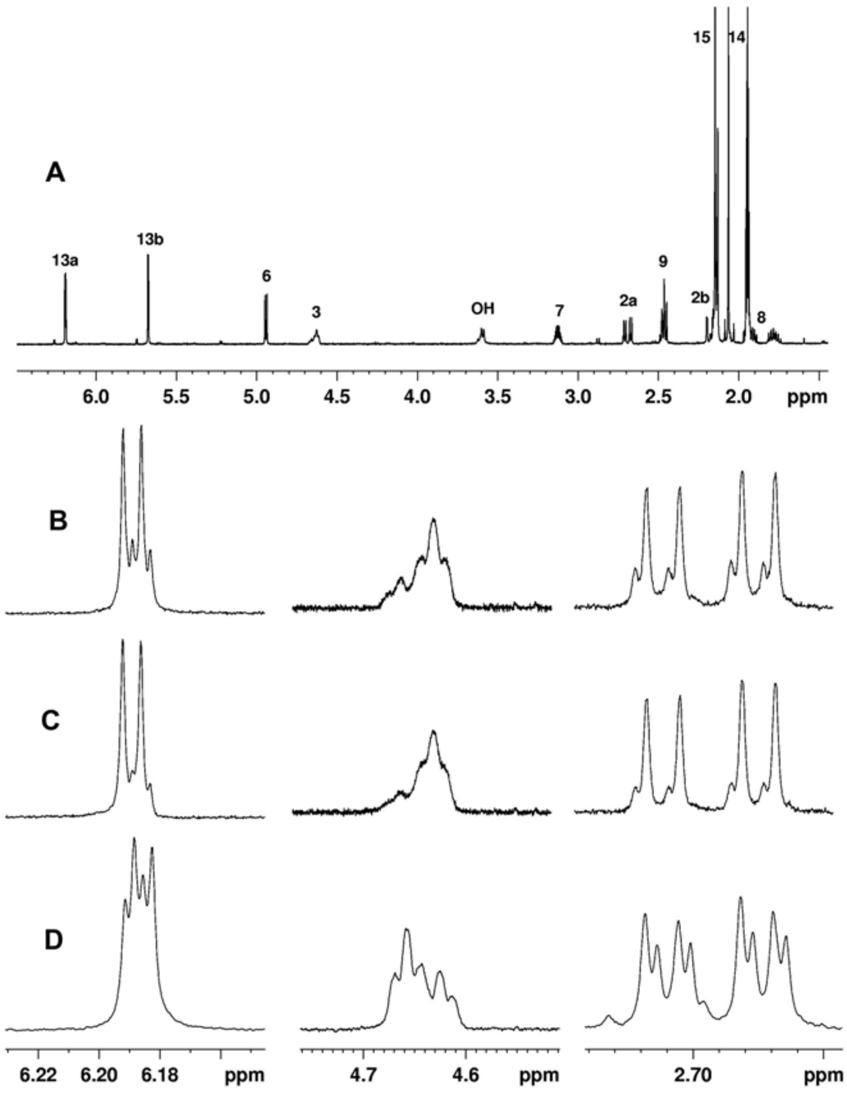

Figure S20. Overlay of ^{1}H NMR spectra (500MHz, $CDCl_3$): A) assigned spectrum of Todorova material isolated from *Achillea*. B) expansion of 3 key regions of the spectrum of Todorova material isolated from *Achillea*. C) sample from B dopped with synthetic 2. D) sample from B dopped with synthetic 1.

Comparison of the optical rotation of Todorova's sample from Achillea ($[\alpha]^{20}_{D}$ = -8.3 (c = 0.0006 in $CHCl_3$) with that of synthetic **1** ($[\alpha]^{20}_{D}$ = +2.9 (c = 0.008 in $CHCl_3$) and synthetic **2**

($[\alpha]^{20}_{D}$ = -6.5 (c = 0.002 in $CHCl_3$) suggested that the major isomer present in Todorova's sample had the same relative and absolute configuration as our synthetic **2** and therefore that **2** is a natural product, which we have named *epi*-*iso*-*seco*-tanapartholide.

During the review process for this manuscript an additional report of the isolation and biological characterisation of the *iso*-*seco*-tanapartholides was published

Ghantous, Akram; Nasser, Niveen; Saab, Ihab; Darwiche, Nadine; Saliba, Najat A.. **Structure-activity relationship of seco-tanapartholides isolated from Achillea falcata for inhibition of HaCaT cell growth.** European Journal of Medicinal Chemistry (2009), 44(9), 3794-3797.

We thank Professor Saliba for forwarding us the ^{1}H NMR spectra ($CDCl_3$, 300MHz) of "compounds 3a and 3b" from this paper. Comparison of these NMR spectra with our authentic samples confirmed that compound 3a has the same relative configuration as *epi*-*iso*-*seco*-tanapartholide **2** and that compound 3b is a mixture of the two epimers, as stated, with *epi*-*iso*-*seco*-tanapartholide **2** being the major one present.

Experimental Procedures

General

Chemicals and solvents were purchased from the Aldrich Chemical Company, Fischer Chemicals, and Alfa Aesar, Lancaster and were used as received unless otherwise stated. Air and moisture sensitive reactions were carried out under an inert atmosphere of dried argon and glassware was oven-dried (145 ^{o}C).

Analytical thin-layer chromatography (TLC) was performed on pre-coated TLC plates SIL G-25 UV_{254} (layer 0.25 mm silica gel with fluorescent indicator UV_{254}) (Aldrich). Developed plates were air-dried and analysed under a UV lamp, Model UVGL-58 (Mineralight LAMP, Multiband $UV_{254/365}$ nm) and where necessary, stained with a solution of potassium permanganate to aid identification. Flash column chromatography was performed using silica gel (40-63 μm) (Fluorochem).

Melting points were determined using an Electrothermal 9100 capillary melting point apparatus. Values are quoted to the nearest 1 ^{o}C and are uncorrected.

^{1}H NMR spectra were recorded on a Bruker Avance 400 (400 MHz) spectrometer. ^{13}C NMR spectra using the PENDANT sequence were recorded on a Bruker Avance 400 (100 MHz) spectrometer. Chemical shifts (δ) are recorded using the residual solvent as the internal reference in all cases ($CDCl_3$ δH 7.27 ppm, δC 77.16 ppm). Coupling constants (J) are quoted to the nearest 0.1Hz. The following abbreviations are used; s, singlet; d, doublet; dd, doublet of doublets; dt, doublet of triplets; t, triplet; m, multiplet and br, broad. Where inseparable mixture of diastereoisomers were obtained, ^{1}H NMR and ^{13}C NMR spectra for the major diastereoisomer only are reported. IR spectra were recorded on a Perkin Elmer Spectrum GX FT-IR spectrometer.

Low resolution and high resolution (HR) electrospray mass spectral (ES-MS) analyses were recorded on a high performance orthogonal acceleration reflecting TOF mass spectrometer, coupled to a Waters 2975 HPLC.

Optical rotation measurements were recorded on a Perkin Elmer 341 polarimeter in the D-line of sodium at 20 ^{o}C using 1 ml solution cell with a 10 cm path length. The concentration (c) is expressed in g/ml.

Experimental protocols

Photolysis of (-)-α-santonin (synthesis of *O*-Acetylisophotosantonin, 12)[S13]

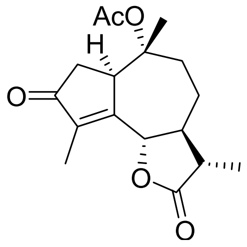

(-)-α-santonin (8.01 g, 32.5 mmol) was dissolved in glacial acetic acid (80 mL) and irradiated using a 125W mercury lamp in a photochemical reactor vessel for 14 hr. The mixture was concentrated and the residual thick brown oil was dissolved in hot methanol (7 mL) and left in a freezer overnight to crystallize. The white crystalline solid product (2.53 g, 25% yield) was collected by filtration and washed with cold methanol (7 mL).

mp 175 – 177 °C (lit. 175 – 177 °C[S13]), **^{1}HNMR** ($CDCl_3$, 400 MHz) 4.79 (1H, d, *J* = 11 Hz, H-6), 4.13 (1H, m, H-1), 2.59 (1H, dt, *J* = 4.5, 13.7, H-8a), 2.50 – 2.26 (3H, m, 2H-2, H-11), 2.22 – 2.13 (2H, m, H-8b, H-7), 2.05 (1H, dtd, *J* = 1.5, 4.1, 14.7 Hz, H-9a), 1.97 (3H, s, CH_3-17), 1.87 (3H, dd, *J* = 1.7, 2.3 Hz, CH_3-14), 1.49 – 1.38 (1H, m, H-9b), 1.25 (3H, d, *J* = 6.8 Hz, CH_3-13), 1.06 (3H, s, CH_3-15).
^{13}CNMR ($CDCl_3$, 100 MHz) 206.9, 177.1, 170.3, 160.9, 143.2, 85.5, 81.2, 48.2, 47.3, 41.3, 37.9, 36.8, 25.3, 22.3, 20.0, 12.4, 9.47 $[\alpha]^{20}_D$ = +48.1 (c = 0.001 in $CHCl_3$, lit. +58, c = 0.53 in EtOH[S13]), **HRMS** (TOF ES^+) (m/z) calcd. for $C_{17}H_{22}0_5Na$ ($[M+Na]^+$): 329.1365, found: 329.1371, **FT-IR** (film) ν 3055, 1783, 1730, 1707, 1422, 1266 cm^{-1}

3-oxo-11β(*S*)*H*-4,10(1)-guaiadien-6α(*S*),12-olide, 8[S14]

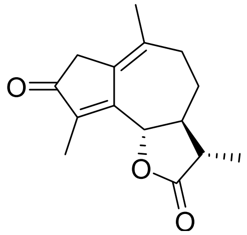

To conc. sulfuric acid (20 mL) at 0°C was added *O*-Acetylisophotosantonic lactone **10** (1.34 g, 4.37 mmol) portionwise over 10 min then stirred for 10 min at 0°C before the ice-bath was removed and the mixture allowed to warm up to room temperature. Stirring was continued for 50 min. Then the resulting brown solution was poured into ice/water mixture and left to warm up to room temperature before extracting with dichloromethane (3 x 60ml). The organic extracts were combined and washed with 5% aqueous sodium hydroxide solution (20 mL), then water (20 mL) and dried (Na_2SO_4) and finally concentrated giving the desired product (1.07 g) as a white solid in quantitative yield. This material was used in subsequent step without further purification. Material should be stored in the dark as it decomposes easily in ordinary light.

mp 87 – 90 °C (lit. 93 – 98 °C recrystallised from 25% methanol-isopropyl ether[S14]), **R_f** = 0.13 (6:4 hexane/ ethyl acetate), **^{1}HNMR** ($CDCl_3$, 400 MHz) 5.21 (1H, d, *J* = 10.5 Hz, H-6), 2.94 (2H, s, H-2),

2.61 (1H, m, H-9a), 2.39 (1H, m, H-11), 2.28 – 2.09 (3H, m, H-8a, 9b, 7), 2.00 (3H, s, CH_3-15), 1.87 (3H, s, CH_3-14), 1.71 (1H, m, H-8b), 1.25 (3H, d, *J* = 6.8 Hz, CH_3-13).
^{13}CNMR ($CDCl_3$, 100 MHz) 204.4, 177.6, 160.8, 139.2, 133.1, 129.5, 80.3, 47.5, 42.3, 40.3, 32.7, 26.9, 24.4, 12.9, 9.81 $[\alpha]^{20}_D$ = +3.1 (c = 0.002 in $CHCl_3$), **HRMS** (TOF ES^+) (m/z) calcd. for $C_{15}H_{18}0_3Na$ ($[M+Na]^+$): 269.1154, found: 269.1159, **FT-IR** (KBr) ν 2976, 2929, 2873, 1781, 1683, 1640, 1595 cm^{-1}

1β(*R*),10β(*S*)-dihydroxy-3-oxo-11β(*S*)*H*-4-guaien-6α(*S*),12-olide, 9 and 1α(*S*),10α(*R*)-dihydroxy-3-oxo-11β(*S*)*H*-4-guaien-6α(*S*),12-olide, 13

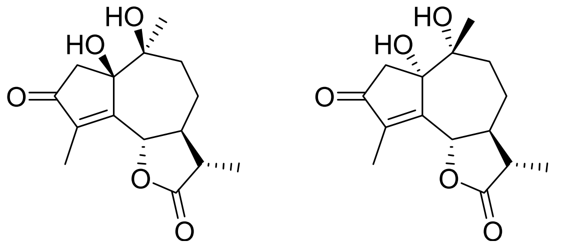

To a solution of alkene **8** (707 mg, 2.87 mmol) and 4-methylmorpholine *N*-oxide (NMO, 681 mg, 5.81 mmol) in 9:1 THF/water (10 mL) at room temperature was added a 2.5 wt % solution of osmium tetroxide in *tert*-butanol (2 mL, 0.160 mmol). The mixture was stirred for 6 hrs and quenched by addition of saturated aqueous sodium sulfite solution (10 ml) and the mixture stirred for 2 hrs before extraction with ethyl acetate (3 x 50 mL) and the combined organic phases were washed with 10% aqueous sodium sulfite solution (10 mL) followed by water (10 mL). After drying, the solvent was removed in vaccuo and the residue was purified by flash column chromatography (SiO2, 4:6 hexanes: ethyl acetate) to give the desired product as a white solid (710 mg, 87%) and 3:1 mixture of inseparable diastereomers. On one occasion, chromatography led to an analytically pure sample of major isomer **9** being isolated.

Major isomer, 9
R_f = 0.14 (4:6 hexane/ ethyl acetate), **^{1}HNMR** ($CDCl_3$, 400 MHz) 5.26 (1H, d, *J* = 10.5 Hz, H-6), 3.96 (1H, s, br, OH), 3.10 (1H, s, br, OH), 2.55 – 2.42 (2H, m, 2H-2), 2.40 – 2.34 (1H, m, H-11), 2.15 – 2.08 (1H, m, H-9a), 1.90 – 1.70 (3H, m, H-8a, 7, 8b), 1.80 (3H, s, CH_3-15), 1.50 – 1.42 (1H, m, H-9b), 1.25 (3H, s, CH_3-14), 1.23 (3H, d, *J* = 7.2 Hz, CH_3-13).
^{13}CNMR ($CDCl_3$, 100 MHz) 205.9, 178.2, 165.5, 137.0, 80.3, 78.2, 73.9, 52.5, 46.6, 42.8, 34.2, 26.1, 23.2, 12.8, 8.43 **HRMS** (TOF ES^+) (m/z) calcd. for $C_{15}H_{20}0_5Na$ ($[M+Na]^+$): 303.1208, found: 303.1210, **FT-IR** (KBr) ν 3448 (br), 2983, 2936, 2879, 1774, 1707, 1650, 1457, 1380 cm^{-1}

1β(*R*),10β(*S*)-bis-(trimethylsilyloxy)-3-oxo-11β(*S*)*H*-4-guaien-6α(*S*),12-olide, 10 and 1α(*S*),10α(*R*)-bis-(trimethylsilyloxy)-3-oxo-11β(*S*)*H*-4-guaien-6α(*S*),12-olide, 14

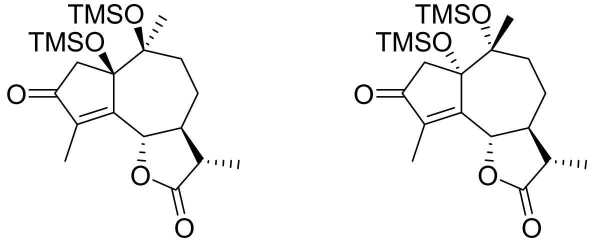

10 **14**

To a solution of mixture of diols **9** and **13** (74 mg, 0.26 mmol) and diisopropylethylamine (0.20 mL 1.2 mmol) in dichloromethane (3 mL) at 0 °C was added TMS-triflate (0.10 mL, 0.55 mmol). The mixture was stirred for 45 min at 0°C then quenched by addition of saturated aqueous sodium bicarbonate solution (6 mL) and product extracted with DCM (3 x 20 ml). The combined organic layers were dried (Na_2SO_4), concentrated and purified by flash column chromatography (SiO_2, 7:3 hexane: ethyl acetate) to give the desired product as a clear oil (99 mg, mixture of diastereomers) in 88% yield. On one occasion, chromatography led to an analytically pure sample of major isomer **10** being isolated.

Major isomer, 10

R_f = 0.36 (7:3 hexane/ ethyl acetate), **^{1}HNMR** ($CDCl_3$, 400 MHz) 5.19 (1H, d, *J* = 10.4 Hz, H-6), 2.61 – 2.37 (2H, m, 2H-2), 2.35 – 2.26 (1H, m, H-11), 2.21 – 2.10 (1H, m, br, H-9a), 1.90 – 1.80 (1H, m, H-8a), 1.83 (3H, s, CH_3-15), 1.79 – 1.62 (2H, m, H-7, H-8b), 1.49 – 1.39 (1H, m, H-9b), 1.23 (3H, d, *J* = 7.2 Hz, CH_3-13), 1.19 (3H, s, CH_3-14), 0.14 (9H, s, $Si(CH_3)_3$), 0.06 (9H, s, $Si(CH_3)_3$).
^{13}CNMR ($CDCl_3$, 100 MHz) 205.4, 177.6, 165.6, 137.3, 83.7, 80.8, 78.5, 51.1, 47.3, 43.2, 34.2, 24.9, 23.8, 12.7, 8.30, 2.58, 1.92 **HRMS** (TOF ES^+) (m/z) calcd. for $C_{21}H_{36}0_5Si_2Na$ ($[M+Na]^+$): 447.1999, found: 447.1993, **FT-IR** (KBr) ν 1786, 1716, 1654, 1458, 1379 cm^{-1}

1β(*R*),10β(*S*)-bis-(trimethylsilyloxy)-3β(*S*)-hydroxy-11β(*S*)*H*-4-guaien-6α(*S*),12-olide, 15 and 1α(*S*),10α(*R*)-bis-(trimethylsilyloxy)-3α(*R*)-hydroxy-11β(*S*)*H*-4-guaien-6α(*S*),12-olide, 16 and 1β(*R*),10β(*S*)-bis-(trimethylsilyloxy)-3α(*R*)-hydroxy-11β(*S*)*H*-4-guaien-6α(*S*),12-olide, 17 and 1α(*S*),10α(*R*)-bis-(trimethylsilyloxy)-3β(*S*)-hydroxy-11β(*S*)*H*-4-guaien-6α(*S*),12-olide, 18

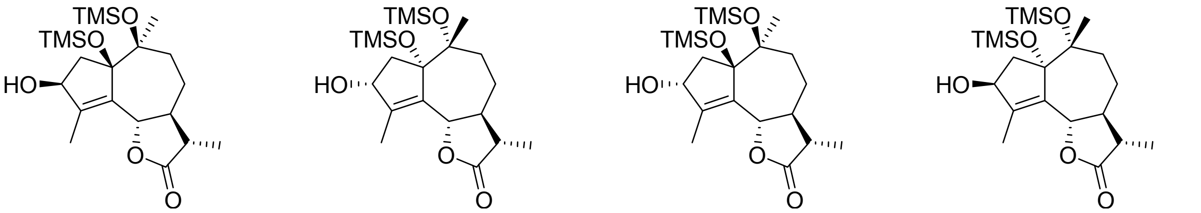

15 **16** **17** **18**

To a solution of mixture of enones **10** and **14** (11.8 g, 27.8 mmol) in methanol (140 mL) at room temperature was added sodium borohydride (1.25 g, 33.0 mmol) portionwise over 50 min. The solution was stirred for further 45 min and quenched by addition of saturated aqueous sodium bicarbonate solution (70 mL) and product extracted with dichloromethane (3 x 150 mL). The combined organic layers were dried (Na_2SO_4), concentrated and purified by flash column chromatography (SiO_2, 4:1 hexane: ethyl acetate) to give the desired products **15** (5.63 g, as a white solid) and **15/16** (3.79 g, as a white solid) and **17** (1.51 g, as white solid) and **17/18** (0.239 g, thick oil) in 95% combined yield. X-ray quality crystals of **15** and **17** were obtained by recrystallisation of small quantities of the pure isolated samples from ethyl acetate/hexane. The fraction containing a mixture of **15** and **16** were repurified by flash column chromatography (SiO_2, 4:1 hexane: ethyl acetate) enabling a small quantity of pure **16** (100mg) to be isolated. X-ray quality crystals of **16** were obtained by recrystallisation of this pure material from diethyl ether/petroleum ether.

For **15** (white solid): **R_f** = 0.38 (7:3 hexane/ ethyl acetate), **mp** 89 – 91 °C, **^{1}HNMR** ($CDCl_3$, 400 MHz) 4.84 (1H, d, *J* = 11 Hz, H-6), 4.29 (1H, m, H-3), 2.80 – 2.74 (1H, dd, *J* = 7.3, 14.3 Hz, H-2a), 2.42 – 2.34 (1H, m, H-9a), 2.29 – 2.21 (1H, m, H-11), 1.91 – 1.81 (1H, m, H-8a), 1.84 (3H, s, CH_3-15), 1.79 – 1.69 (2H, m, H-7, OH), 1.75 – 1.69 (1H, dd, *J* = 4.9, 14.6 Hz, H-2b), 1.54 – 1.40 (2H, m, H-8b, H-9b), 1.20 (3H, d, *J* = 7.0 Hz, CH_3-13), 1.04 (3H, s, CH_3-14), 0.14 (9H, s, $Si(CH_3)_3$), 0.13 (9H, s, $Si(CH_3)_3$).
^{13}CNMR ($CDCl_3$, 100 MHz) 178.6, 138.6, 137.8, 89.8, 80.2, 78.2, 78.0, 49.8, 45.3, 44.3, 33.6, 24.1, 23.4, 13.0, 12.0, 2.96, 2.71 $[\alpha]^{20}_D$ = -72.2 (c = 0.003 in $CHCl_3$), **HRMS** (TOF ES^+) (m/z) calcd. for

$C_{21}H_{38}0_5Si_2Na$ ($[M+Na]^+$): 449.2156, found: 449.2144, **FT-IR** (KBr) ν 3462, 2948, 2883, 1754, 1713, 1686, 1639 cm^{-1}

For **16** (white solid): $\mathbf{R_f}$ = 0.32 (7:3 hexane/ ethyl acetate), **mp** 159 – 160 °C, **^{1}HNMR** ($CDCl_3$, 400 MHz) 4.44 (1H, d, *J* = 11 Hz, H-6), 4.39 – 4.31 (1H, m, H-3), 3.10 – 3.01 (1H, dd, *J* = 8.1, 15.7 Hz, H-2a), 2.78 – 2.67 (1H, dq, *J* = 1.3, 11.2 Hz, H-9a), 2.33 – 2.23 (1H, dt, *J* = 4.2, 13.5 Hz, H-8a), 2.19 – 2.08 (1H, m, H-11), 1.87 (3H, s, CH_3-15), 1.86 – 1.79 (1H, m, H-7), 1.83 – 1.77 (1H, dd, *J* = 4.2, 15.7 Hz, H-2b), 1.63 – 1.54 (2H, m, H-9b, OH), 1.27 – 1.10 (1H, m, H-8b), 1.22 (3H, d, *J* = 7.0 Hz, CH_3-13), 0.86 (3H, s, CH_3-14), 0.13 (9H, s, $Si(CH_3)_3$), 0.12 (9H, s, $Si(CH_3)_3$).
^{13}CNMR ($CDCl_3$, 100 MHz) 179.2, 146.9, 134.7, 93.1, 81.5, 81.0, 76.9, 45.5, 45.1, 41.8, 38.4, 24.8, 23.5, 13.1, 12.6, 2.93, 2.58 $[\alpha]^{20}_D$ = +59.0 (c = 0.001 in $CHCl_3$), **HRMS** (TOF ES^+) (m/z) calcd. for $C_{21}H_{38}0_5Si_2Na$ ($[M+Na]^+$): 449.2156, found: 449.2151, **FT-IR** (KBr) ν 3459, 2912, 2881, 1761, 1723, 1672, 1637 cm^{-1}

For **17** (white solid): $\mathbf{R_f}$ = 0.19 (7:3 hexane/ ethyl acetate), **mp** 130 °C, **^{1}HNMR** ($CDCl_3$, 400 MHz) 5.01 (1H, d, *J* = 9.0 Hz, H-6), 4.72 – 4.65 (1H, m, H-3), 2.28 – 2.21 (1H, dd, *J* = 6.3, 13.0 Hz, H-2a), 2.25 – 2.16 (1H, m, H-9a), 1.85 (3H, s, CH_3-15), 1.77 – 1.59 (6H, m, H-9b, H-8a, H-8b, H-11, H-7, OH), 1.55 – 1.48 (1H, dd, *J* = 6.3, 13.0 Hz, H-2b), 1.28 (3H, s, CH_3-14), 1.22 (3H, d, *J* = 7.0 Hz, CH_3-13), 0.16 (9H, s, $Si(CH_3)_3$), 0.05 (9H, s, $Si(CH_3)_3$).
^{13}CNMR ($CDCl_3$, 100 MHz) 179.1, 142.0, 138.5, 88.4, 79.4, 79.0, 77.1, 54.8, 47.9, 42.9, 35.5, 26.1, 24.9, 12.8, 11.5, 2.69, 2.23 $[\alpha]^{20}_D$ = -12.8 (c = 0.0026 in $CHCl_3$), Anal. ($C_{21}H_{38}0_5Si_2$) **C, H, N**. calculated %C 59.11, %H 8.98, analysed: %C 59.37, %H 9.36 **FT-IR** (KBr) ν 3468, 2953, 2887, 1754, 1709, 1684, 1643 cm^{-1}

18 was not isolated pure enough to enable characterization.

1β(*R*),10β(*S*)-bis-(trimethylsilyloxy)-3β(*S*)-(*tert*-butyldimethylsilyloxy)-11β(*S*)*H*-4-guaien-6α(*S*),12-olide, 19 and 1α(*S*),10α(*R*)-bis-(trimethylsilyloxy)-3α(*R*)-(*tert*-butyldimethylsilyloxy)-11β(*S*)*H*-4-guaien-6α(*S*),12-olide, 20

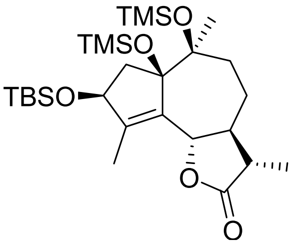

19

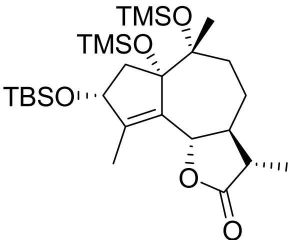

20

To a solution of mixture of alcohols **15** and **16** (522 mg, 1.22 mmol) and diisopropylethylamine (1.10 mL, 6.31 mmol) in dichloromethane (20 mL) at 0°C was added TBS-triflate (0.530 mL, 2.31 mmol). The reaction mixture was stirred at 0°C for 30 min then allowed to warm up to room temperature and stirred for a further 70 min. Quenching was by addition of saturated aqueous sodium bicarbonate solution (20 mL) and product extracted with DCM (3 x 60 mL). The combined organic layers were dried (Na_2SO_4), concentrated and the residue purified by flash column chromatography (SiO_2, 9:1 hexane: ethyl acetate) to give the desired products (**19** – 391 mg and **20** – 203 mg) in 90% combined yield.

For **19:**

$\mathbf{R_f}$ = 0.32 (9:1 hexane/ ethyl acetate), **^{1}HNMR** ($CDCl_3$, 400 MHz) 4.79 (1H, d, *J* = 10.8 Hz, H-6), 4.26 (1H, dd, *J* = 5.3, 7.8 Hz, H-3), 2.74 – 2.67 (1H, dd, *J* = 7.5, 14.5 Hz, H-2a), 2.50 – 2.40 (1H, m, H-9a), 2.29 – 2.19 (1H, m, H-11), 1.93 – 1.71 (3H, m, H-8a, H-2b, H-7), 1.76 (3H, s, CH_3-15), 1.52 – 1.38 (2H, m, H-8b, H-9b), 1.20 (3H, d, *J* = 7.0 Hz, CH_3-13), 1.00 (3H, s, CH_3-14), 0.90 (9H, s, $SiC(CH_3)_3$), 0.13 (9H, s, $Si(CH_3)_3$), 0.12 (9H, s, $Si(CH_3)_3$), 0.070 (3H, s, $SiCH_3$), 0.067 (3H, s, $SiCH_3$).
^{13}CNMR ($CDCl_3$, 100 MHz) 178.6, 139.2, 135.9, 89.9, 80.3, 78.2, 77.8, 49.4, 45.2, 44.4, 33.4, 25.9, 24.1, 23.4, 18.2, 13.0, 12.0, 2.96, 2.47, -4.24, -4.78 $[\alpha]^{20}_D$ = -46.6 (c = 0.017 in $CHCl_3$), **HRMS** (TOF ES^+) (m/z) calcd. for $C_{27}H_{52}0_5Si_3Na$ ($[M+Na]^+$): 563.3020, found: 563.3022

For **20**:
$\mathbf{R_f}$ = 0.23 (9:1 hexane/ ethyl acetate), **^{1}HNMR** ($CDCl_3$, 400 MHz) 4.42 (1H, d, *J* = 11.2 Hz, H-6), 4.31 – 4.25 (1H, dd, *J* = 3.7, 7.9, H-3), 3.00 – 2.93 (1H, dd, *J* = 8.3, 15.6 Hz, H-2a), 2.75 – 2.65 (1H, ddd, *J* = 11.8, 17.5, 23.1 Hz, H-7), 2.34 – 2.25 (1H, dt, *J* = 4.05, 13.5 Hz, H-9a), 216 – 2.06 (1H, m, H-11), 1.83 – 1.74 (2H, m, H-8a, H-2b), 1.76 (3H, s, CH_3-15), 1.58 – 1.51 (1H, td, *J* = 3.4, 13.2 Hz, H-9b), 1.20 (3H, d, *J* = 6.9 Hz, CH_3-13), 1.17 – 1.11 (1H, td, *J* = 3.3, 14.1 Hz, H-8b), 0.89 (9H, s, $SiC(CH_3)_3$), 0.82 (3H, s, CH_3-14), 0.11 (9H, s, $Si(CH_3)_3$), 0.09 (9H, s, $Si(CH_3)_3$), 0.07 (3H, s, $SiCH_3$), 0.06 (3H, s, $SiCH_3$).
^{13}CNMR ($CDCl_3$, 100 MHz) 179.2, 147.8, 132.8, 93.1, 81.6, 81.1, 76.6, 45.6, 45.5, 41.7, 38.3, 25.9, 24.8, 23.6, 18.1, 13.3, 12.5, 2.91, 2.32, -4.22, -4.81 $[\alpha]^{20}_D$ = +56.6 (c = 0.008 in $CHCl_3$), **HRMS** (TOF ES^+) (m/z) calcd. for $C_{27}H_{52}0_5Si_3Na$ ($[M+Na]^+$): 563.3020, found: 563.3024

1β(*R*),10β(*S*)-bis-(trimethylsilyloxy)-3β(*S*)-(*tert*-butyldimethylsilyloxy)-11β(*R*)-phenylseleno-4-guaien-6α(*S*),12-olide, 21

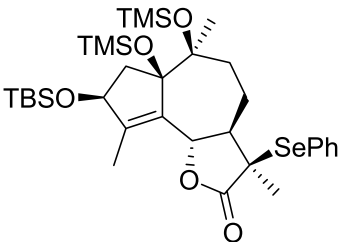

To a solution of lactone **19** (322 mg, 0.595 mmol) in THF (5 mL) at -78°C was added 1.0M THF solution of lithium bis(trimethylsilyl)amide (LiHMDS, 1.80 mL, 1.80 mmol) and mixture stirred at this temperature for 80 min then a solution of diphenyldiselenide (245 mg, 0.785 mmol) and HMPA (0.140 mL, 0.805 mmol) in THF (3 mL) was added. The resulting mixture was stirred at -78°C for a further 50 min and warmed up to -40°C and stirring continued for 1.5 hrs before reaction was quenched by addition 0.1M aqueous HCl solution (12 mL) and product extracted with ethyl acetate (120 mL). The organic phase was washed with brine, dried (Na_2SO_4) and concentrated. The residue was purified by flash column chromatography (SiO_2, 9:1 hexane: ethyl acetate) to give the desired product (352 mg) as a pale yellow solid in 85% yield.

$\mathbf{R_f}$ = 0.28 (9:1 hexane/ ethyl acetate), **mp** 44 – 46°C, **^{1}HNMR** ($CDCl_3$, 400 MHz) 7.66 – 7.62 (2H, m, -Se*Ph*), 7.44 – 7.39 (1H, tt, *J* = 2.1, 7.4 HZ, -Se*Ph*), 7.35 – 7.30 (2H, m, -Se*Ph*), 4.98 (1H, d, *J* = 10.7 Hz, H-6), 4.28 – 4.23 (1H, t, *J* = 6.2 Hz, H-3), 2.74 – 2.66 (1H, dd, *J* = 7.5, 14.4 Hz, H-2a), 2.45 – 2.35 (1H, m, H-9a), 2.10 – 1.92 (2H, m, H-7, H-8a), 1.82 – 1.70 (2H, m, H-2b, H-8b), 1.75 (3H, s, CH_3-15), 1.49 (3H, s, CH_3-13), 1.48 – 1.44 (1H, m, H-9b), 1.01 (3H, s, CH_3-14), 0.90 (9H, s, $SiC(CH_3)_3$), 0.16 (9H, s, $Si(CH_3)_3$), 0.15 (9H, s, $Si(CH_3)_3$), 0.07 (6H, s, $Si(CH_3)_2$).
^{13}CNMR ($CDCl_3$, 100 MHz) 175.9, 139.8, 138.4, 135.6, 129.9, 129.2, 124.7, 89.7, 80.2, 77.8, 76.8, 53.6, 53.5, 45.4, 34.1, 25.9, 23.5, 22.3, 20.9, 18.1, 12.2, 3.04, 2.53, -4.25, -4.79 $[\alpha]^{20}_D$ = -11.1 (c =

0.007 in $CHCl_3$), **HRMS** (TOF ES^+) (m/z) calcd. for $C_{33}H_{56}0_5Si_3SeNa$ ($[M+Na]^+$): 719.2499, found: 719.2493, **FT-IR** (KBr) ν 3069, 2942, 2846, 1774, 1568, 1361, 1289 cm^{-1}

1β(*R*),10β(*S*)-bis-(trimethylsilyloxy)-3β(*S*)-(*tert*-butyldimethylsilyloxy)-4,11(13)-guaiadien-6α(*S*),12-olide, 22

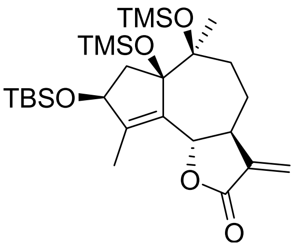

To a solution of α-phenylseleno lactone **21** (352 mg, 0.506 mmol) in THF (2.50 mL) at 0 °C were added successively glacial acetic acid (80.0 μl) and 30% hydrogen peroxide solution (0.350 mL). The reaction mixture was stirred for 35 min at 0 °C and then quenched by addition of saturated aqueous sodium bicarbonate solution (10 mL) dropwise and product extracted with ether (2 x 50 mL), dried and concentrated. The residue was then dissolved in DCM (50 mL) and washed with brine (10 mL), dried and concentrated to give the desired product (260 mg, 96% yield) as a clear oil in high purity such that this material was used in the subsequent step without need for further purification.

R_f = 0.27 (9:1 hexane/ ethyl acetate), **^{1}HNMR** ($CDCl_3$, 400 MHz) 6.18 (1H, d, *J* = 3.7 Hz, H-13a), 5.42 (1H, d, *J* = 3.4 Hz, H-13b), 4.76 (1H, d, *J* = 10.7 Hz, H-6), 4.31 – 4.26 (1H, t, *J* = 6.3 Hz, H-3), 2.80 – 2.73 (1H, dd, *J* = 7.4, 14.6 Hz, H-2a), 2.73 – 2.65 (1H, m, H-7), 2.55 – 2.45 (1H, m, H-9a), 2.11 – 1.99 (1H, m, H-8a), 1.81 – 1.75 (1H, m, H-2b), 1.79 (3H, s, CH_3-15), 1.70 – 1.61 (1H, m, H-9b), 1.53 – 1.46 (1H, m, H-8b), 1.02 (3H, s, CH_3-14), 0.91 (9H, s, $SiC(CH_3)_3$), 0.13 (9H, s, $Si(CH_3)_3$), 0.12 (9H, s, $Si(CH_3)_3$), 0.08 (6H, s, $Si(CH_3)_2$).
^{13}CNMR ($CDCl_3$, 100 MHz) 170.2, 141.5, 139.5, 135.6, 119.4 89.8, 80.4, 78.0, 77.8, 45.7, 45.1, 32.9, 25.9, 23.1, 23.0, 18.2, 12.0, 2.99, 2.43, -4.22, -4.76 $[\alpha]^{20}_D$ = -86.8 (c = 0.008 in $CHCl_3$), CI-**HRMS** (m/z) calcd. for $C_{27}H_{51}0_5Si_3$ ($[M+H]^+$): 539.3044, found: 539.3032, **FT-IR** (KBr) ν 2957, 2857, 1771, 1257 cm^{-1}

1β(*R*),3β(*S*),10β(*S*)-trihydroxy-4,11(13)-guaiadien-6α(*S*),12-olide, 7

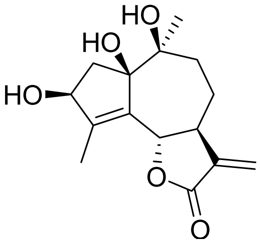

To a solution of fully silyl-protected triol **22** (639 mg, 1.19 mmol) in dry THF (15 mL) at 0°C was added TBAF (8.00 mL, 1.0 M in THF, 8.00 mmol). The resulting solution was stirred at 0°C for 1.5 hrs then allowed to warm up to room temperature and stirred for a further 1.5 hrs at which point saturated aqueous ammonium chloride solution (20 mL) was added and mixture extracted with ethyl acetate (2 x 100 mL). Combined organic layers were washed with brine (40 mL), dried ($NaSO_4$) and concentrated under vacuum. The residue was purified by flash column chromatography (SiO_2, ethyl acetate) to provide the desired product (289 mg) as a white solid in 87% yield.

R_f = 0.17 (ethyl acetate), **mp** 44 °C, **^{1}HNMR** ($CDCl_3$, 400 MHz) 6.03 (1H, d, *J* = 3.5 Hz, H-13a), 5.51 (1H, d, *J* = 3.3 Hz, H-13b), 4.99 – 4.95 (1H, dd, *J* = 0.6, 10.4 Hz, H-6), 4.31 – 4.27 (1H, dd, *J* = 2.5, 6.8 Hz, H-3), 4.15 (1H, br s, OH), 3.95 (1H, br s, OH), 3.77 (1H, br s, OH), 2.73 – 2.64 (1H, m, H-7), 2.46 – 2.39 (1H, dd, *J* = 7.8, 15.1 Hz, H-2a), 2.13 – 1.97 (2H, m, H-8a, H-9a), 1.90 – 1.75 (1H, dtd, *J* = 1.5, 4.0, 14.5 Hz, H-9b), 1.88 (3H, s, CH_3-15), 1.73 – 1.67 (1H, dd, *J* = 3.4, 15.0 Hz, H-2b), 1.59 – 1.51 (1H, m, H-8b), 1.15 (3H, s, CH_3-14).
^{13}CNMR (Acetone-d_6, 75 MHz) 169.7, 141.8, 140.0, 138.9, 118.1, 86.9, 78.8, 77.6, 74.6, 49.3, 45.1, 34.2, 24.8, 22.5, 12.1 $[\alpha]^{20}_D$ = -90.5 (c = 0.0075 in acetone), **HRMS** (TOF ES^+) (m/z) calcd. for $C_{15}H_{20}0_5Na$ ($[M+Na]^+$): 303.1208, found: 303.1212, **FT-IR** (KBr) ν 3402, 2986, 2941, 2867, 1777, 1759, 1676 cm^{-1}

***iso-seco*-tanapartholide, 1**

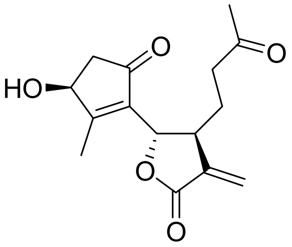

To a solution of triol **7** (38 mg, 0.13 mmol) in dry acetone (3 mL) at 0°C was added lead tetraacetate (119 mg, 0.27 mmol) and stirred for 30 min at 0°C. The solvent was removed in vaccuo and the residue purified by flash column chromatography (SiO_2, ethyl acetate) to provide the desired product (32 mg) as a clear oil in 86% yield.

R_f = 0.17 (ethyl acetate), **^{1}HNMR** ($CDCl_3$, 400 MHz) 6.30 (1H, d, *J* = 2.8 Hz, H-13a), 5.64 (1H, d, *J* = 2.5 Hz, H-13b), 4.90 (1H, d, *J* = 5.3 Hz, H-6), 4.73 – 4.67 (1H, t, *J* = 6.1 Hz, H-3), 3.28 (1H, d, *J* = 7.2 Hz, OH), 3.15 – 3.08 (1H, m, H-7), 2.79 – 2.71 (1H, dd, *J* = 6.3, 18.6 Hz, H-2a), 2.56 – 2.48 (2H, m, 2H-9), 2.32 – 2.26 (1H, dd, *J* = 2.1, 18.6 Hz, H-2b), 2.15 (3H, s, CH_3-15), 2.12 (3H, s, CH_3-14), 2.02 – 1.77 (2H, m, 2H-8).
^{13}CNMR ($CDCl_3$, 100 MHz) 208.0, 203.6, 173.6, 170.3, 138.4, 137.3, 122.9, 76.5, 71.7, 44.4, 42.7, 39.7, 30.1, 27.5, 14.2 $[\alpha]^{20}_D$ = +2.9 (c = 0.008 in $CHCl_3$), **HRMS** (TOF ES^+) (m/z) calcd. for $C_{15}H_{18}0_5Na$ ($[M+Na]^+$): 301.1052, found: 301.1046, **FT-IR** (KBr) ν 2921, 1758, 1705, 1654, 1383, 1277, 1144 cm^{-1}

3-*O*-Acetyl-*iso*-*seco*-tanapartholide, 3

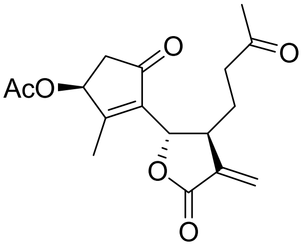

The mixture of *iso*-*seco*-tanapartholide **1** (22 mg, 0.079 mmol), pyridine (0.70 mL) and acetic anhydride (0.40 mL) at room temperature was stirred for 6 hrs then poured into a separating funnel, diluted with ethyl acetate (50 mL) and washed with 10% aqueous HCl solution (20 mL) then saturated brine (5 mL). The organic phase was dried (Na_2SO_4), filtered and concentrated. The residue was purified by flash

column chromatography (SiO_2, 1:1 hexanes: ethyl acetate) to give the desired product (25 mg) as an oil in quantitative yield.

R_f = 0.44 (ethyl acetate), **^{1}HNMR** ($CDCl_3$, 400 MHz) 6.36 (1H, d, *J* = 2.6 Hz, H-13a), 5.68 (1H, d, *J* = 2.4 Hz, H-13b), 5.69 – 5.66 (1H, br m, H-3), 4.99 (1H, d, *J* = 4.9 Hz, H-6), 3.17 – 3.09 (1H, m, H-7), 2.92 – 2.84 (1H, dd, *J* = 6.4, 18.9 Hz, H-2a), 2.66 – 2.49 (2H, m, 2H-9), 2.33 – 2.26 (1H, dd, *J* = 2.1, 18.9 Hz, H-2b), 2.16 (3H, s, CH_3-15), 2.13 (3H, s, CH_3-14), 2.10 (3H, s, CH_3-17), 2.00 – 1.85 (2H, m, 2H-8).
^{13}CNMR ($CDCl_3$, 100 MHz) 207.5, 202.2, 170.6, 169.8, 168.8, 140.0, 138.1, 123.3, 76.2, 73.1, 43.1, 41.8, 39.5, 30.2, 27.7, 21.0, 14.5 $[\alpha]^{20}_D$ = +17.2 (c = 0.0025 in $CHCl_3$), **HRMS** (TOF ES^+) (m/z) calcd. for $C_{17}H_{20}0_6Na$ ($[M+Na]^+$): 343.1158, found: 343.1162, **FT-IR** (KBr) ν 1754, 1730, 1703, 1661, 1379, 1234 cm^{-1}

1α(*S*),10α(*R*)-bis-(trimethylsilyloxy)-3α(*R*)-(*tert*-butyldimethylsilyloxy)-11β(*R*)-phenylseleno-4-guaien-6α(*S*),12-olide, 23

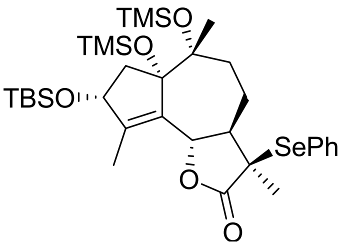

To a solution of lactone **20** (1.39 g, 2.57 mmol) in THF (10 mL) at -78°C was added 1.0M THF solution of lithium bis(trimethylsilyl)amide (LiHMDS, 7.50 mL, 7.50 mmol) and mixture stirred at this temperature for 80 min then a solution of diphenyldiselenide (1.21 g, 3.88 mmol) and HMPA (0.500 mL, 2.87 mmol) in THF (5 mL) was added. The resulting mixture was stirred at -78°C for a further 50 min and warmed up to -40°C and stirring continued for 1.5 hrs before reaction was quenched by addition 0.1M aqueous HCl solution (40 mL) and product extracted with ethyl acetate (350 mL). The organic phase was washed with brine, dried (Na_2SO_4) and concentrated. The residue was purified by flash column chromatography (SiO_2, 9:1 hexane: ethyl acetate) to give the desired product (1.53 g) as a white crystalline solid in 85% yield.

R_f = 0.41 (9:1 hexane/ ethyl acetate), **mp** 106 – 108°C, **^{1}HNMR** ($CDCl_3$, 400 MHz) 7.67 – 7.63 (2H, m, -Se*Ph*), 7.43 – 7.38 (1H, tt, *J* = 2.1, 7.4 HZ, -Se*Ph*), 7.35 – 7.29 (2H, m, -Se*Ph*), 4.77 (1H, d, *J* = 11.2 Hz, H-6), 4.31 – 4.26 (1H, m, H-3), 3.04 – 2.93 (2H, m, H-7, H-2a), 2.34 – 2.24 (1H, dt, *J* = 4.0, 13.5 Hz, H-9a), 1.89 – 1.82 (1H, dtd, *J* = 1.3, 4.0, 14.5 Hz, H-8a), 1.81 – 1.75 (1H, dd, *J* = 4.2, 15.5 Hz, H-2b), 1.72 (3H, s, CH_3-15), 1.65 – 1.58 (1H, td, *J* = 3.7, 13.1 Hz, H-9b), 1.50 (3H, s, CH_3-13), 1.38 –

1.26 (1H, m, H-8b), 0.90 (9H, s, SiC(CH_3)$_3$), 0.83 (3H, s, CH_3-14), 0.13 (9H, s, Si(CH_3)$_3$), 0.09 (9H, s, Si(CH_3)$_3$), 0.08 (6H, s, Si(CH_3)$_2$).
^{13}CNMR ($CDCl_3$, 100 MHz) 176.5, 148.5, 138.3, 132.5, 129.7, 129.2, 124.7, 93.1, 81.5, 78.7, 76.6, 51.5, 49.3, 45.7, 38.4, 25.9, 23.6, 23.1, 22.3, 20.9, 18.1, 13.2, 2.95, 2.36, -4.21, -4.79 $[\alpha]^{20}_D$ = +83.1 (c = 0.004 in $CHCl_3$), **HRMS** (TOF ES$^+$) (m/z) calcd. for $C_{33}H_{56}0_5Si_3SeNa$ ([M+Na]$^+$): 719.2499, found: 719.2505, **FT-IR** (KBr) ν 3060, 2957, 2858, 1772, 1579, 1376, 1251 cm^{-1}

1α(*S*),10α(*R*)-bis-(trimethylsilyloxy)-3α(*R*)-(*tert*-butyldimethylsilyloxy)-4,11(13)-guaiadien-6α(*S*),12-olide, 24

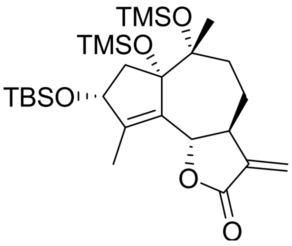

To a solution of α-phenylseleno lactone **23** (487 mg, 0.700 mmol) in THF (4 mL) at 0 °C were added successively glacial acetic acid (100 μl) and 30% hydrogen peroxide solution (0.500 mL). The reaction mixture was stirred for 35 min at 0 °C and then quenched by addition of saturated aqueous sodium bicarbonate solution (12 mL) dropwise and product extracted with ether (2 x 60 mL), dried and concentrated. The residue was then dissolved in DCM (70 mL) and washed with brine (10 mL), dried and concentrated to give the desired product (355 mg, 94% yield) as a clear oil in high purity such that this material was used in the subsequent step without need for further purification.

R_f = 0.42 (9:1 hexane/ ethyl acetate), **^{1}HNMR** ($CDCl_3$, 400 MHz) 6.15 (1H, d, *J* = 3.4 Hz, H-13a), 5.42 (1H, d, *J* = 3.1 Hz, H-13b), 4.40 (1H, d, *J* = 11.2 Hz, H-6), 4.32 – 4.27 (1H, m, H-3), 3.68 – 3.60 (1H, m, H-7), 3.01 – 2.94 (1H, dd, *J* = 8.2, 15.6 Hz, H-2a), 2.42 – 2.32 (1H, dt, *J* = 4.2, 13.4 Hz, H-9a), 2.09 – 2.01 (1H, dtd, *J* = 1.3, 4.0, 14.5 Hz, H-8a), 1.86 – 1.80 (1H, dd, *J* = 3.9, 15.5 Hz, H-2b), 1.81 (3H, s, CH_3-15), 1.64 – 1.57 (1H, td, *J* = 3.5, 13.1 Hz, H-9b), 1.29 – 1.13 (1H, m, H-8b), 0.91 (9H, s, SiC(CH_3)$_3$), 0.83 (3H, s, CH_3-14), 0.13 (9H, s, Si(CH_3)$_3$), 0.11 (9H, s, Si(CH_3)$_3$), 0.09 (3H, s, Si(CH_3)$_2$), 0.08 (3H, s, Si(CH_3)$_2$).
^{13}CNMR ($CDCl_3$, 100 MHz) 170.8, 148.2, 140.0, 132.4, 117.9, 93.3, 81.7, 81.4, 76.7, 45.5, 42.4, 37.9, 25.9, 23.7, 23.6, 18.2, 13.5, 2.96, 2.44, -4.19, -4.80 $[\alpha]^{20}_D$ = +77.7 (c = 0.001 in $CHCl_3$), **HRMS** (TOF ES$^+$) (m/z) calcd. for $C_{27}H_{50}0_5Si_3Na$ ([M+Na]$^+$): 561.2864, found: 561.2866, **FT-IR** (KBr) ν 2958, 2859, 1774, 1253 cm^{-1}

1α(*S*),3α(*R*),10α(*R*)-trihydroxy-4,11(13)-guaiadien-6α(*S*),12-olide, 25

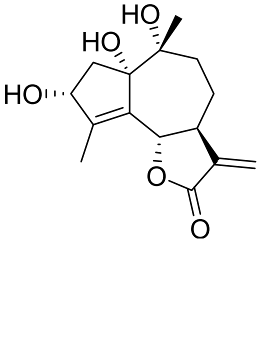

To a solution of fully silyl-protected triol **24** (752 mg, 1.40 mmol) in dry THF (10 mL) at 0°C was added TBAF (10.0 mL, 1.0 M in THF, 10.0 mmol). The resulting solution was stirred at 0°C for 1.5 hrs then allowed to warm up to room temperature and stirred for a further 1.5 hrs at which point saturated aqueous ammonium chloride solution (20 mL) was added and mixture extracted with ethyl acetate (2 x 100 mL). Combined organic layers were washed with brine (40 mL), dried ($NaSO_4$) and concentrated under vacuum. The residue was purified by flash column chromatography (SiO_2, ethyl acetate) to provide the desired product (360 mg) as a white solid in 92% yield.

R*f* = 0.17 (ethyl acetate), **mp** 154 – 155 °C, **^{1}HNMR** (Acetone-*d*$_6$, 400 MHz) 6.07 (1H, d, *J* = 3.5 Hz, H-13a), 5.57 (1H, d, *J* = 3.2 Hz, H-13b), 4.72 – 4.67 (1H, td, *J* = 1.4, 11.2 Hz, H-6), 4.38 – 4.32 (1H, br d, *J* = 7.0 Hz, H-3), 4.05 (1H, br s, OH), 3.81 – 3.72 (1H, m, H-7), 3.43 (1H, br s, OH), 3.10 – 3.03 (1H, dd, *J* = 7.8, 15.1 Hz, H-2a), 2.92 (1H, br s, OH), 2.39 – 2.30 (1H, dt, *J* = 4.4, 13.7 Hz, H-9a), 2.21 – 2.14 (1H, dtd, *J* = 1.5, 4.0, 14.5 Hz, H-8a), 1.92 (3H, s, CH_3-15), 1.73 – 1.67 (1H, dd, *J* = 3.4, 15.0 Hz, H-2b), 1.68 – 1.62 (1H, td, *J* = 3.6, 13.4 Hz, H-9b), 1.44 – 1.31 (1H, m, H-8b), 0.95 (3H, s, CH_3-14).
^{13}CNMR (Acetone-*d*$_6$, 100 MHz) 170.1, 148.0, 141.1, 134.4, 117.4, 90.7, 81.7, 76.8, 76.4, 47.0, 43.0, 38.8, 23.9, 22.1, 13.3 $[\alpha]^{20}_D$ = +142.5 (c = 0.002 in acetone), Anal. ($C_{15}H_{20}0_5$) **C, H, N**. calculated %C 64.27, %H 7.19, analysed: %C 64.31, %H 7.28, **FT-IR** (KBr) ν 3405, 2987, 2942, 2865, 1778, 1757, 1675 cm^{-1}

3-*epi*-*iso*-*seco*-tanapartholide, 2

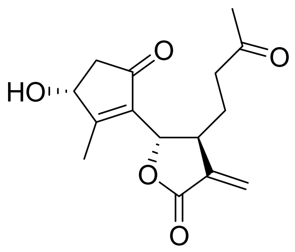

To a solution of triol **25** (167 mg, 0.596 mmol) in 20 mL of dry DCM/Acetone (3:1) at 0°C was added lead tetraacetate (326 mg, 0.735 mmol) and stirred for 25 min at 0°C. Then a small amount of silica was added into the flask and solvent was removed under vacuum. The residue was dry loaded onto column and purified by flash column chromatography (SiO_2, ethyl acetate) to provide the desired product (163 mg) as a thick oil in 98% yield.

R*f* = 0.17 (ethyl acetate), **^{1}HNMR** ($CDCl_3$, 400 MHz) 6.33 (1H, d, *J* = 2.8 Hz, H-13a), 5.65 (1H, d, *J* = 2.4 Hz, H-13b), 4.94 (1H, d, *J* = 5.3 Hz, H-6), 4.70 (1H, t, *J* = 5.4 Hz, H-3), 3.13 – 3.05 (1H, m, H-7), 2.98 (1H, br d, *J* = 6.8 Hz, OH), 2.83 – 2.74 (1H, dd, *J* = 6.3, 18.6 Hz, H-2a), 2.62 – 2.46 (2H, m, 2H-9), 2.35 – 2.28 (1H, dd, *J* = 2.2, 18.6 Hz, H-2b), 2.17 (3H, s, CH_3-15), 2.14 (3H, s, CH_3-14), 2.01 – 1.80 (2H, m, 2H-8).
^{13}CNMR ($CDCl_3$, 100 MHz) 207.9, 203.4, 173.3, 170.2, 138.4, 137.7, 123.0, 76.1, 71.7, 44.4, 42.9, 39.6, 30.1, 27.4, 14.2 $[\alpha]^{20}_D$ = -6.5 (c = 0.002 in $CHCl_3$), **HRMS** (TOF ES$^+$) (m/z) calcd. for $C_{15}H_{18}0_5Na$ ($[M+Na]^+$): 301.1052, found: 301.1053, **FT-IR** (KBr) ν 2924, 1760, 1706, 1655, 1384, 1276, 1142 cm^{-1}

Biology experimental procedures

a) **IC_{50} Determination in Hela 57A assay (TNFα-stimulated production of a luciferase reporter gene).**
Hela 57As were seeded at 3000 cells per well in each well of a 96 well plate (Greiner) in D-Mem containing 10% foetal calf serum and standard antibiotics. The cells were left to grow at 37^0C for 3 days after which time compound was added as a solution in DMSO. For each dose response curve generated, cells were treated with compound at a final concentration of 100, 50, 25, 12.5, 6.25, 3.12, 1.56 and 0 μM for 2 hrs. For each compound six dose response curves were generated per analysis. After the two hours preincubation with compound was complete, a solution of TNFα in D-Mem (10ng/ml) was added to each well and the plate incubated at 37^0C for a further 5 hrs. The media was then carefully removed from each well and the cells washed with PBS (2 x 50μl). The final wash was removed and luciferase lysis buffer (100μl/well) was then added to each well and the plate incubated at room temperature for 20 minutes. A solution of 100 μl luciferase assay buffer (containing luciferin) was then added and the plate read on plate luminometer giving a readout in Relative Light Units(RLU) for each well. IC_{50} values were determined using SigmaPlot® software. Data for each compound was plotted and a curve of best fit was applied to each data set (R^2= at least 0.98). The equation for each best fit curve was used to calculate the IC_{50} (the reported values are the average of at least 3 calculated values).

b) **Western blot analysis**: Experiments were carried out according to the protocol previously reported by Arenzana-Seisdedos, Hay *et al.*[S15]

c) **Band shift assays:** Experiments were carried out according to the protocol previously reported by Matthews, Hay *et al.*[S16] As shown in Figures S21 and S22, a clear dose dependent response was observed when these experiments were carried out in the presence of either **1** or **2**. As was the case for the IC_{50} determinations, **1** and **2** are essentially equipotent in this assay.

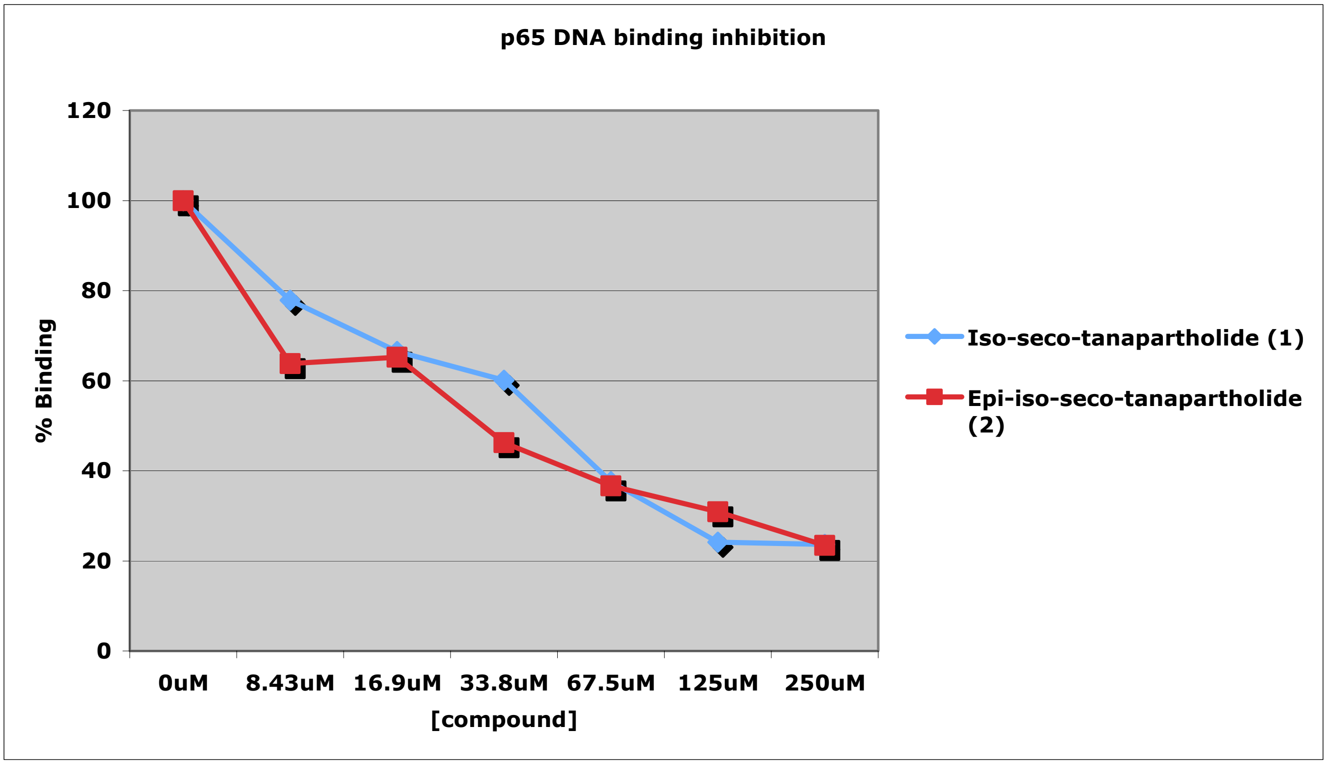

Figure S21. Analysis of **1** and **2** in the p65 band shift assay

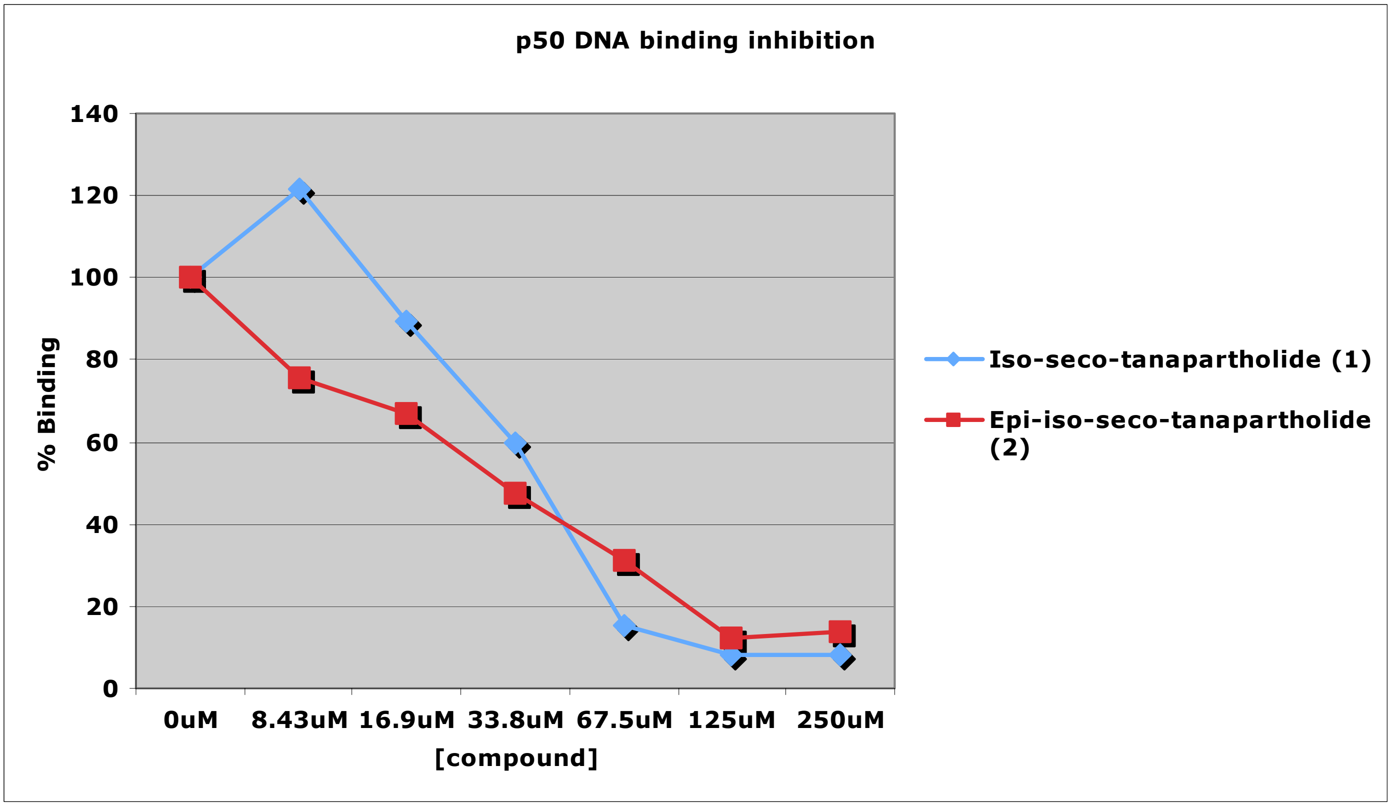

Figure S22. Analysis of **1** and **2** in the p50 band shift assay

d) **Immunofluorescence experiments:**

As discussed, further evidence to support the mode of action of our natural product extract was carried out using immunofluorescence based experiments that probed the location of the p65-subunit of NF-κB. Again, due to limitations on material, this experiment was carried out using fraction 8 of the extract #2335. The experiment was carried out with nasal polyps fibroblast cells as follows: Eosinophils (2.5 x 10^6/ml) were cultured at 37ºC in Iscove´s DMEM containing 5% FCS for 2 hours. The cells were centrifuged and dried on air for 10 minutes. The cells were then fixed with 4% (w/v) p-formaldehyde/PBS for 10 minutes and washed 3 times with PBS. The cells were permeabilized and non-specific binding was blocked in buffer containing 0.2% (w/v) Triton X-100 in DAKO Protein block Serum Free buffer at room temperature for 30 minutes. Rabbit polyclonal p65 antibody diluted in DAKO Antibody Diluent with 0.2% (v/v) Triton X-100 was added to the cells for 1 hour. The cells were washed 3 times with the same buffer and incubated with anti-mouse IgG FITC antibody, diluted in DAKO Antibody Diluent with 0.2% (v/v) Triton X-100, for 1 hour. Finally, cells were washed 3 times in the same buffer, glass coverslip were applied and cells examined by fluorescent microscope.

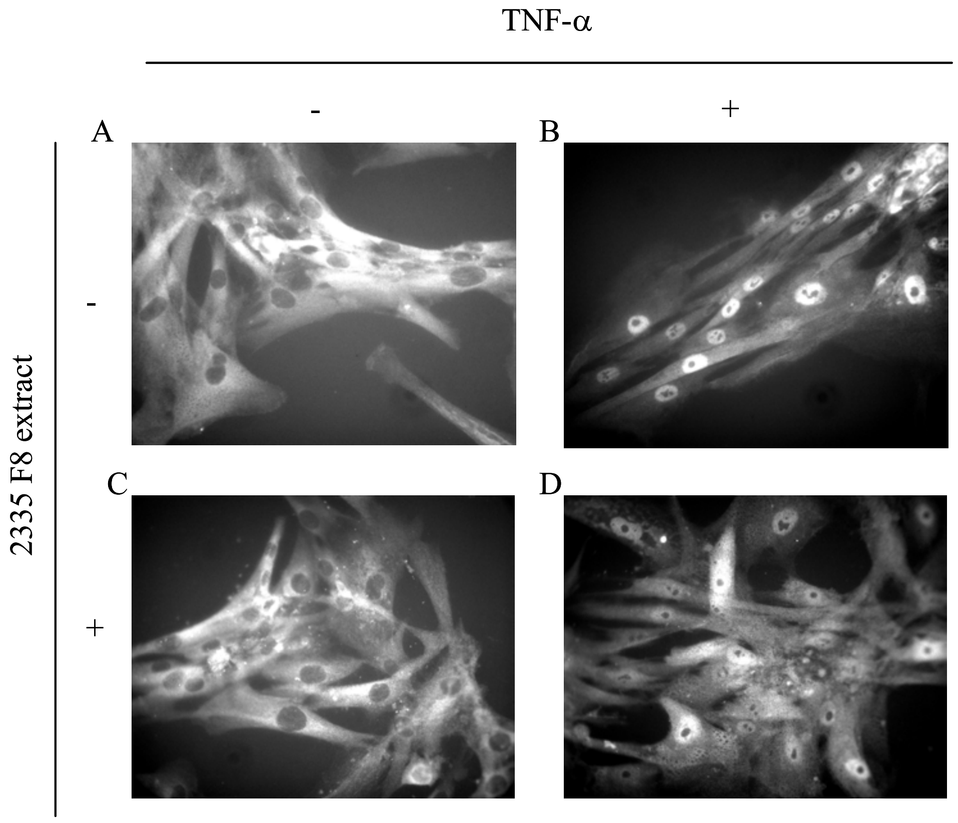

Figure S23. Immunofluorescence using nasal polyps fibroblasts cells, stimulated with TNF-α. A, cells were not pre-treated and not stimulated. B, cells were not pre-treated but stimulated with 50ng/ml TNF-α. C, cells were pre-treated with 20,0x10^{-2} mg/ml of 2335 F8 extract and stimulated with 50ng/ml TNF-α after 2 hours incubation. D, cells were pre-treated with 20,0x10^{-2} mg/ml of 2335 F8 extract, but not stimulated. Coverslips were fixed and immunoblotted with the rabbit polyclonal p65 antibody.

References

S1 Rodriguez, M.S.; Thompson, J.; Hay, R.T.; Dargemont, C., *J. Biol. Chem.*, **1999**, *274(13)*, 9108.

S2 Kwok, B.H.B.; Koh, B.; Ndubuisi, M.I.; Elofsson, M.; Crews, C.M., *Chemistry & Biology*, **2001**, *8(8)*, 759.

S3 Huneck, S.; Zdero, C.; Bohlmann, F. *Phytochemistry*, **1986**, *25*, 883.

S4 Marco, J.A.; Sanz-Cervera, J.F.; Manglano, E.; Sancenon, F.; Rustaiyan, A.; Kardar, M. *Phytochemistry*, **1993**, *34*, 1561.

S5 Jakupovic, J.; Chen, Z.L.; Bohlmann, F., *Phytochemistry*, **1987**, *26(10)*, 2777.

S6 Tan, R.X.; Jakupovic, J.; Bohlmann, F.; Jia, Z.J.; Huneck, S., *Phytochemistry*, **1991**, *30(2)*, 583.

S7 Marco, J.A.; Sanz-Cervera, J.F.; Garcia-Lliso, V.; Batlle, N., *Phytochemistry*, **1997**, *45(4)*, 755.

S8 Todorova, M.N.; Tsankova, E.T.; Taskova, R.M.; Peev, D.R., *Journal of Biosciences*, **1999**, *54(12)*, 1011.

S9 Todorova, M.; Tsankova, E., *Zeitschrift fuer Naturforschung, C: Journal of Biosciences* **2001**, 56(11/12), 957.

S10 Ahn, H.; Kim, J.Y.; Lee, H.J.; Kim, Y.K.; Ryu, J.-H., *Archives of Pharmaceutical Research*, **2003**, *26(4)*, 301.

S11 Todorova, M.; Mustakerova, E.; Tsankova, E., *Dokladi na Bulgarskata Akademiya na Naukite*, **2005**, *58(1)*, 25.

S12 Todorova, M., Ognyanov, I., *Planta Medica*, **1985**, *51(2)*, 174

S13 Barton, D.H.R.; De Mayo, P.; Shafiq, M. *J. Chem. Soc.,* **1957**, 929.

S14 Metz, P.; Bertels, S.; Frohlich, R. *J. Am. Chem. Soc.,* **1993**, *115*, 12595.

S15 Arenzana-Seisdedos, F.; Turpin, P.; Rodriguez, M.; Thomas, D.; Hay, R.T.; Virelizier, J.-L.; Dargemont, C., *Journal of Cell Science*, **1997**, *110(3)*, 369.

S16 Matthews J R; Kaszubska W.; Turcatti G.; Wells T.N.; Hay R.T., *Nucleic Acids Res.*, **1993**, *21(8)*, 1727.
